# Injectable and 3D-Printable Semi-Interpenetrating Polymer Networks Based on Modified Sodium Alginate for Cell Spheroid Formation

**DOI:** 10.1021/acs.biomac.4c01343

**Published:** 2024-12-30

**Authors:** Sofia Falia Saravanou, Thomai Samouilidou, Constantinos Tsitsilianis, Stavros Taraviras, George Pasparakis

**Affiliations:** † Department of Chemical Engineering, University of Patras, Patras 26504, Greece; ‡ Department of Physiology, School of Medicine, University of Patras, Patras 26504, Greece

## Abstract

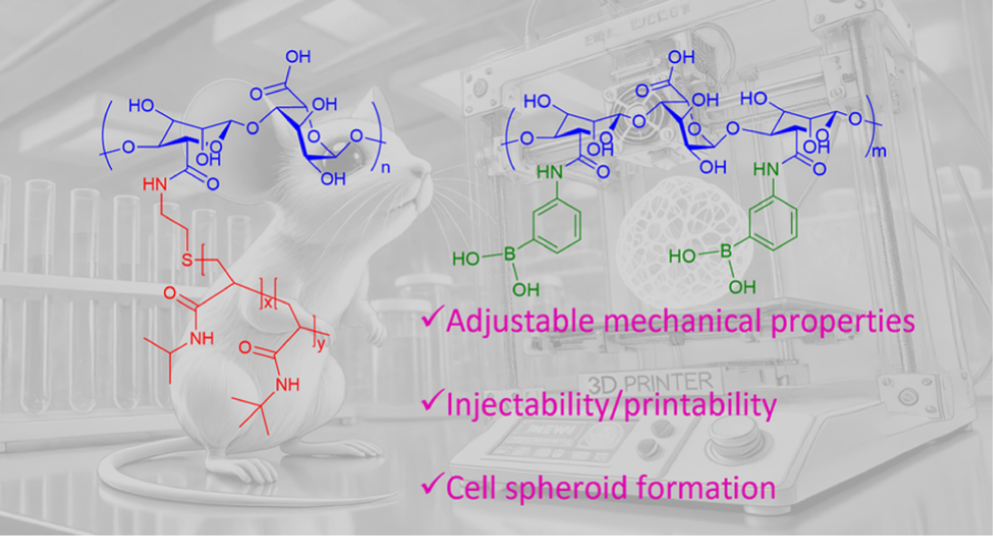

We report on 3D-printable polymer networks based on the combination of modified alginate-based polymer blends; two alginate polymers were prepared, namely, a thermoresponsive polymer grafted with P(NIPAM_86_-*co*-NtBAM_14_)-NH_2_ copolymer chains and a second polymer modified with diol/pH-sensitive 3-aminophenylboronic acid. The gelation properties were determined by the hydrophobic association of the thermosensitive chains and the formation of boronate esters. At a mixing ratio of 70/30 wt % of the thermo/diol-responsive polymers, the semi-interpenetrating network exhibited an optimum storage modulus ranging from ca. 150 Pa at 20 °C up to ca. 480 Pa at 37 °C due to the stimulated cross-linking synergism. The resulting bioink blends could promote the rapid formation of cell spheroids with an average diameter of 62.5 μm within 24 h. The network could easily be dissociated by the addition of free glucose, acting as an antagonistic disruptor of the cross-links. The proposed material was found to be nontoxic, with adequate injectability and 3D printability.

## 1 Introduction

Semi-interpenetrating polymer networks (SIPNs) represent a novel class of systems that have gained increasing attention for their ability to enhance and amplify various material properties, such as stimuli-responsiveness, mechanical strength, and processability.^1−5^ Interpenetrating polymer networks consist of two or more polymers that are not covalently bonded to each other; however, SIPNs are distinguished by the cross-linking of at least one of the polymer networks.^6^

The semi-interpenetration approach has been used to develop multifunctional hydrogel blends that combine the properties of their constituent polymer parts, resulting in polymer networks with tunable properties and enhanced versatility in terms of functionality and application. These multifunctional materials, often referred to as “smart” materials, have been explored for use in drug delivery systems, tissue engineering, and regenerative medicine. Of great interest are alginate-based SIPNs that have been reported by combining synthetic or natural biopolymers with applications in wound healing, cell encapsulation, structural biomaterial design, controlled drug delivery, and self-healing matrices for biomedical applications.

Alginate has been previously reported as a cell encapsulation matrix to form 3D spheroids, as it constitutes a cytocompatible, nonadherent substrate that better promotes cell–cell interactions in a 3D cell culture microenvironment. For example, alginate hydrogels have been used to encapsulate hepatocytes to form spheroids that better mimic liver tissue, showing improved functionality compared to 2D cultures.^7^ Similarly, alginate-based matrices have been employed to encapsulate tumor cells, enabling the formation of tumor spheroids that more closely resemble the in vivo tumor microenvironment, making them ideal for drug screening studies.^8,9^

Boronic acids constitute interesting moieties in the formation of smart polymer networks owing to their unique covalent bond formation with *cis*-diol residues in a pH-dependent manner. Boronate esters have been employed as reversible cross-link segments for reversible polymer network formation or as carbohydrate-anchoring moieties for controlled release applications (i.e., for diabetes management). Interestingly, boronic acids can act as specific cell membrane glycoprotein receptors, which have resulted in several reports on cell-sorting applications, cell encapsulation technologies, and cell-targeting approaches (i.e., cancer cells that overexpress certain glycoproteins or the targeting of the glycocalyx of bacterial cells).

We have previously reported on dynamic self-healable polymer networks that function as cellular glues, as well as simple polymer structures capable of inducing cell aggregate formation with potential applications in cell therapies.^10^ These materials interact with living cells and the structural components of their polymer networks, adding functional complexity that can mimic the extracellular matrix and recapitulate the biological environment of living tissues.^11−13^ Furthermore, the dynamic (de)crosslinking across the macromolecular scaffold suggests that harnessing scaling laws could enhance mechanical properties beyond the additive contributions of the individual polymers.^14^ By blending different polymers, synergism can be fine-tuned through simple adjustment of mixing ratios, leading to significant improvements in bulk mechanical performance.

**Scheme 1:**
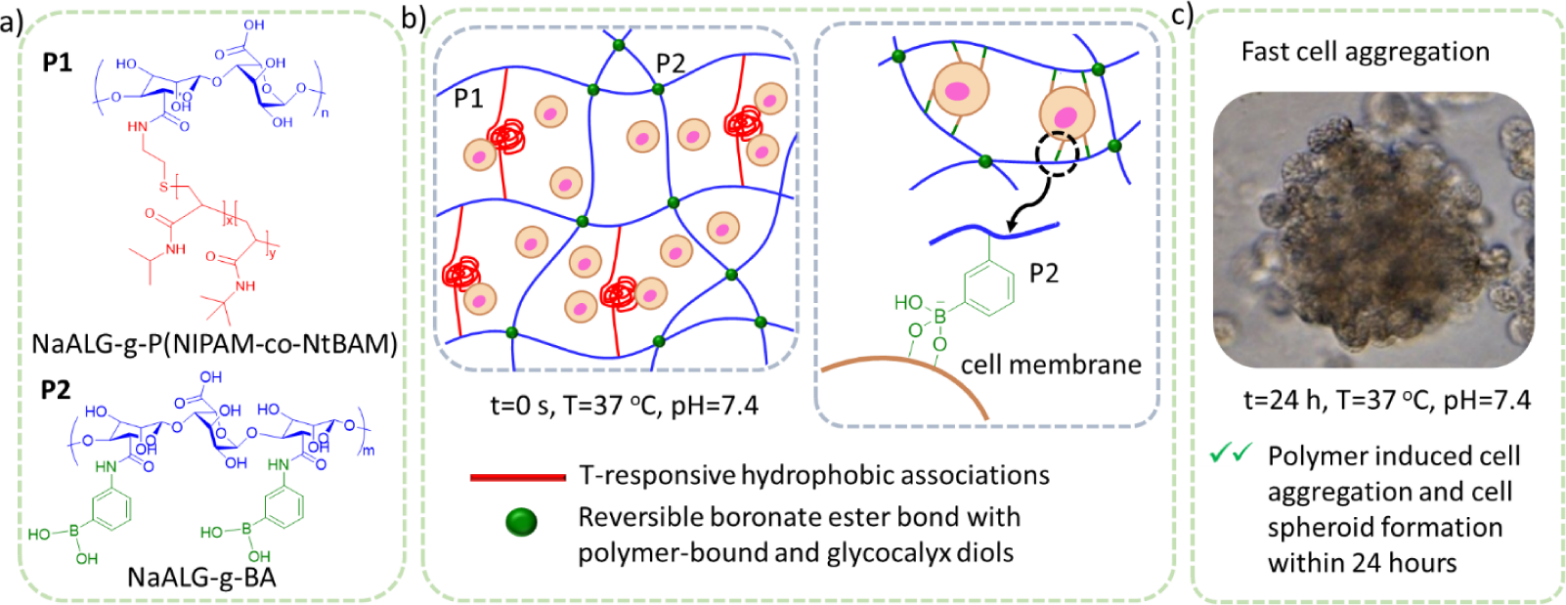
a) The Structure of the Copolymers Used to Form the SIPNs; b) Crosslinking Mechanisms and Interactions with HEK 293T Cells Resulting in c) Rapid Cell Spheroid Formation

To this end, we embarked on the development of a unique blend network based on two alginate-based copolymers with different functional groups, namely one with grafted side chains of poly(*N*-isopropylacrylamide) enriched with the hydrophobic comonomer *N*-tertiary-butyl-acrylamide ([NaALG-*g*-P(NIPAM-*co*-NtBAM)])^15,16^ and another functionalized with the diol-responsive 3-aminophenylboronic acid and [NaALG-*g*-BA] (Scheme 1). Our proposed design rationale leads to simultaneous sensitivity against temperature and diols (i.e., carbohydrates, glycans, etc.) and broad tunability of the dynamic mechanical properties of the polymer matrix, enabling 3D-printability under mild conditions.

The gelation of the polymer blend comprises two distinct, albeit interconnected, mechanisms that we have studied systematically. At room temperature, the boronate ester formation between the BA stickers and the diols of mannuronic/guluronic acid unit blocks of alginate-based backbones occurs, while heating up to physiological body temperature (above the LCST of the PNIPAM-based side chains), the network is reinforced by additional hydrophobic associations of the T-responsive stickers, which, in turn, have a measurable effect on the dissociation of the boronate esters. Furthermore, the BA moieties form boronate esters with the cell-surface glycans when cells are introduced into the polymer matrix, leading to the rapid formation of well-structured cell aggregates (Scheme 1). The simple addition of glucose can easily disrupt network formation by blocking the BA interaction with polymer-bound diols. More interestingly, different mixing ratios induce the formation of completely different networks in terms of synergistic effects on their overall mechanical performance and 3D-printability, highlighting the simplicity of the approach toward the formation of highly tunable polymer networks.

## 2 Experimental Section

### 2.1 Materials

3-Aminophenylboronic acid hydrochloride (BA, Aldrich), *N*-hydroxysuccinimide (NHS, Fluorochem), 1-ethyl-3-(3-(dimethylamino)propyl)carbodiimide (EDC, Alfa Aesar), 2-morpholinoethanesulfonic acid (MES, Fluorochem), d-(+)-glucose (Glucose, Sigma-Aldrich), resazurin sodium salt (Cayman), Dulbecco’s modified Eagle’s medium high-glucose (DMEM, Biosera), fetal bovine serum (FBS, PAN-Biotech), penicillin–streptomycin solution (P/S, Biosera), and trypsin-ethylenediaminetetraacetic acid (Trypsin-EDTA, Biosera) were used as purchased. Purified triple-distilled water (3D-H_2_O) was provided by an ELGA Medica-R7/15 device. NaALG-g_1_-P(NIPAM_86_-*co*-NtBAM_14_) was used as received based on a synthetic method described elsewhere.^15,17^

### 2.2 Synthesis of the NaALG-*g*-BA Graft

For the synthesis of the NaALG-*g*-BA polymer, 1.5 g (7.57 mmol) of NaALG was dissolved in 50 mL of 0.1 M MES aqueous buffer solution, with pH fixed at 5.5 utilizing 1 M HCl, as described in the preparation protocol by Hong et al.^18^ The mixture was left stirring at room temperature overnight. Then, 0.4379 g (2.5252 mmol) of BA, 1.0214 g (5.3282 mmol, 2.11% moles over the BA moieties) of EDC, and 0.1460 g (1.2683 mmol, 0.5% moles over the BA moieties) of NHS were added to the mixture. The final aqueous solution was stirred until full homogeneity was reached for 24 h at room temperature. The resulting material was purified against 3D-H_2_O through a dialysis membrane (MWCO, 3500 Da) for 48 h and was later obtained as a solid by lyophilization.

### 2.3 ^1^H NMR Characterization

^1^H NMR characterization of NaALG-*g*-BA was performed using a BRUKER AVANCE III HD PRODIGY ASCEND 600 MHz spectrometer at room temperature in D_2_O.

### 2.4 Fourier-Transform Infrared Spectroscopy

The FTIR characterization of the NaALG-*g*-BA powder was performed at room temperature using a Nicolet 6700 FTIR spectrometer equipped with a diffuse reflectance (DRIFT) cell (Spectra Tech), an MCT detector, and a KBr beam splitter.

### 2.5 Hydrogel Formation

Five wt % NaALG-*g*-P(NIPAM_86_-*co*-NtBAM_14_)/NaALG-*g*-BA blend polymers were dissolved in 3D-H_2_O, and the aqueous solvents were left under stirring at 200 rpm until full homogeneity was achieved at 20 °C. Specifically, each polymer was weighed separately according to the desired wt % ratio, i.e., NaALG-*g*-P(NIPAM_86_-*co*-NtBAM_14_)/NaALG-*g*-BA at mixing ratios of 30/70 wt %, 50/50 wt %, and 70/30 wt %. The lyophilized polymers were placed together in glass vials and were diluted with water up to a final hydrogel concentration of 5 wt %. The pH of the polymer hydrogels was adjusted to 7.4 by using 1 M NaOH.

### 2.6 Rheological Studies

The thermal and shear sensitivities of the NaALG-based SIPN gels were investigated via a stress-controlled AR-2000ex (TA Instruments) rheometer with a cone-plate geometry (diameter: 20 mm, angle: 3°, and truncation: 111 μm). The setup is equipped with an external trap to limit any concentration changes. The SIPN hydrogels were loaded onto the plate, which maintained the experimental temperature constant (±0.1 °C). All experiments were conducted in the linear viscoelastic regime (LVR), evaluated by shear sweep tests at an angular frequency of 6.28 rad/s.

### 2.7 Scanning Electron Microscopy (SEM) Analysis

5 wt % SIPN, NaALG-*g*-P(NIPAM_86_-*co*-NtBAM_14_), and NaALG-*g*-BA aqueous hydrogels were prepared as described previously. Polymer samples were either heated at 37 °C or at room temperature in order to form gels, which were immediately frozen in liquid nitrogen, followed by lyophilization. The freeze-dried samples were sputter-coated with gold and examined using a scanning electron microscope (SEM, LEO-SURRA VP35 with EDX Microanalysis Unit, Bruker). Three images were analyzed for each material, and the average pore size of the samples was estimated by measuring 150 pores in each photograph using ImageJ software.

### 2.8 Cell Culture

The human embryonic kidney cell line (HEK 293T) was incubated at 37 °C under 5% CO_2_ in Dulbecco’s modified Eagle’s medium enriched with 10% fetal bovine serum and 1% P/S. Every 48 h, when the cells were 90% confluent, they were recovered.

### 2.9 Cell Viability Assay

The resazurin sodium salt assay was utilized for quantitative evaluation of the HEK 293T cell viability. First, the solid polymers and the resazurin powder were sterilized under UV light for 30 min. 20 × 10^3^ cells were embedded gently in 48-well plates within 200 μL of 5 wt % NaALG-*g*-P(NIPAM_86_-*co*-NtBAM_14_)/NaALG-*g*-BA in DMEM, and the systems were incubated for 24 and 48 h at 37 °C under a constant flow of 5% CO_2_. Cells incubated only in DMEM were used as a control. Next, the polymer blend was removed and replaced with 200 μL of 2% v/v resazurin sodium salt/DMEM solution and placed into the incubator for 3.5 h. The blue resazurin dye solution was reduced to pink resorufin due to the metabolic activity in the mitochondria of the living cells. After 3.5 h, the absorbance of the supernatant was measured at 570 and 600 nm in a Multiskan SkyHigh Microplate spectrophotometer (Thermo Fisher Scientific).^19,20^ The cell viability was evaluated by eq 1 as the percentage of viability of the hydrogel-treated cells over the untreated ones:
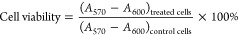
1

### 2.10 3D Printing

A DIY commercial Ender 3 Neo 3D printer was converted into a potential 3D bioprinter in the lab, as proposed by Samokhin’s syringe pump setup.^21^ The 3D-printed designs were created using the free software Tinkercad, and the 3D printer was controlled by PrusaSlicer software. The dimensions of the 3D printed squares were 2.5 cm × 2 cm × 0.1 cm, i.e., five layers of 0.2 mm each. 2 mL of the 5 wt % NaALG-*g*-P(NIPAM86-*co*-NtBAM14)/NaALG-*g*-BA aqueous blend were loaded into a 5 mL Luer lock syringe bearing a 25G needle with an inner diameter of 0.26 mm. The syringe was attached to the printer’s modified extruder. The 3D printing parameters were as follows: i. the extrusion velocity (*Z*-axis) was set at 250 mm/s, ii. the bed velocity in *X* and *Y* axes was set at 25 mm/s, and iii. the hydrogels were extruded on a heated bed at 37 °C. The measurements were evaluated by ImageJ, and the hydrogel samples were dyed with blue food coloring for better visual observation.

## 3 Results and Discussion

The NaALG-*g*-P(NIPAM-*co*-NtBAM) graft copolymer was prepared as reported previously,^15,17^ while the NaALG-*g*-BA copolymer was synthesized via carbodiimide coupling of the amine groups of 3-aminophenylboronic acid with the carboxyl groups of mannuronic/guluronic unit blocks of the sodium alginate backbone. The molar composition (mol/mol) of the grafting BA stickers onto the sodium alginate backbone was evaluated from the ^1^H NMR spectrum by integrating the area at 7.3–7.7 ppm, i.e., representative of the four protons of the phenyl ring of BA^18^ over the 3.5–4.6 ppm, which corresponds to the four protons of the NaALG ring (Figure S1).^22^ The weight composition (w/w) of the NaALG-*g*-BA was determined by the molar composition by utilizing the *M*_w_ of M/G acid blocks of NaALG and the *M*_w_ BA stickers. The grafting density of the BA stickers over the NaALG backbone and the molecular characteristics of the final polymer are presented in Table 1; the molecular weight of the material was calculated by eq 2 as follows:
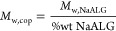
2where *M*_w,NaALG_ = 140 000.^15^

**Table 1:** Molecular Characteristics of NaALG-BA

Polymer	*M*_w_ (g/mol)^a^	%Weight Composition NaAlg/BA (w/w)	%mol Composition NaALG/BA (mol/mol)	Grafting Density^b^
NaALG-g-BA	180,900	77.4/22.6	75/25	177

a Calculated by eq 2.

b Number of grafted BA stickers per sodium alginate backbone by ^1^H NMR.

Next, the successful grafting was evaluated by FTIR. The spectrum of the as-received 3-aminophenylboronic acid was first recorded as a reference (Figure S2) with the area at 900–800 cm^–1^ assigned to the C=C stretching of the BA phenyl ring. The band at 1350 cm^–1^ corresponds to the B–O stretching of BA, which is seen in the spectrum of the pregrafted BA stickers and the NaALG-*g*-BA spectrum. The peak at 3305 cm^–1^ is assigned to the NH_2_ group of the starting BA derivative, which is absent in the spectrum of NaALG-*g*-BA due to its conversion to amide bonds. The latter amide I and II bands can be seen in the 1600–1700 cm^–1^ area, indicative of successful BA coupling with the carboxylate groups of NaALG.

**Figure 1:**
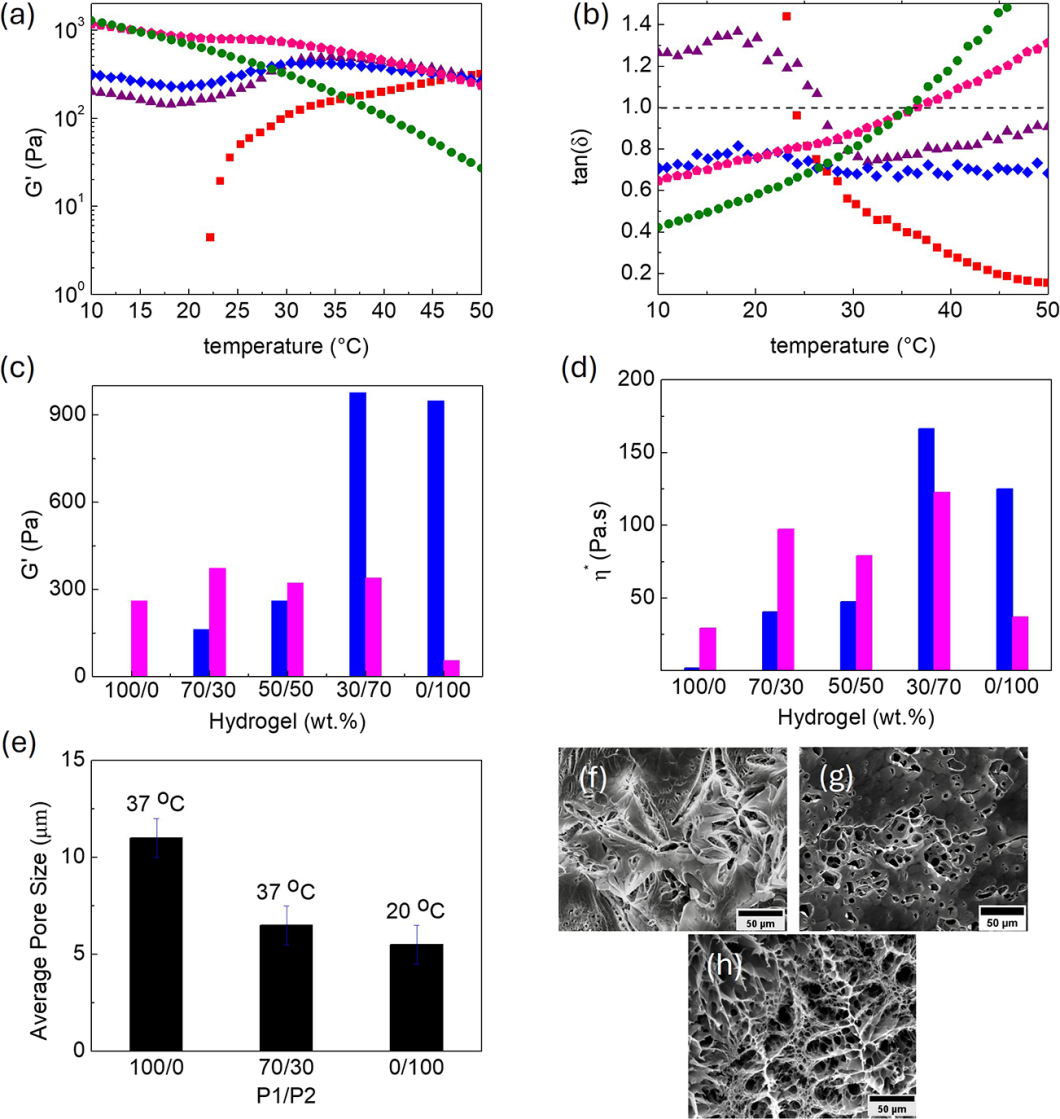
(a) Storage modulus and (b) tan(δ) as a function of temperature of different 5 wt % P1/P2 hydrogels at various blending ratios, i.e., 100/0 (red squares), 70/30 (purple triangles), 50/50 (blue rhombi), 30/70 (pink pentagons), and 0/100 (olive circles) at a frequency of 1 Hz, strain amplitude 0.1% during the heating cycle with a heating rate of 1 °C/min; (c) storage modulus at various blending ratios at 15 °C (low *T* region, blue), and 45 °C (high *T* region, magenta); (d) complex viscosity of the SIPNs at 20 °C (blue) and 37 °C (magenta) in the same heating cycle. (e) Average pore size diameter of P1 and P1/P2 = 70/30 at 37 °C and P2 at 20 °C; SEM images of (f) P1 at 37 °C, (g) P1/P2 = 70/30 at 37 °C, and (h) P2 at 20 °C scaffold (scale bar: 50 μm).

After the development of the proposed materials, oscillatory shear rheology revealed the thermosensitive capacity of the SIPN hydrogels that consisted of NaALG-*g*-P(NIPAM_86_-*co*-NtBAM_14_) (P1) and NaALG-*g*-BA (P2). The networks were studied at three blending ratios (wt %) i.e., P1/P2: 70/30, 50/50, and 30/70. For the sake of comparison, the properties of the blends were correlated with the individual polymer constituents P1 and P2. Figure 1a,b demonstrates the response of the formulations, in terms of storage modulus (*G*′) and loss factor (tan δ), on applying a heating ramp with a rate of 1 °C/min. The overall thermal behavior of the formulations is presented in Figure S3, including heating–cooling cycles, displaying negligible hysteresis as the formed networks are fully reversible. Concerning the pure constituents of the blends, we notice that P1 displays an abrupt increase of *G*′ with temperature, exhibiting a thermo-induced sol–gel transition at *T*_gel_ = 24 °C, due to the activation of the intermolecular hydrophobic association of the LCST-type thermosensitive PNIPAM-based pendants.^25^ On the other hand, P2 exhibits a continuous decrease of *G*′, reaching a gel–sol transition at *T*_sol_= 37 °C, due to possible thermosensitivity of the reversible boronate ester bonds. As shown in Figure 1b, these two opposite effects are reflected in the blends, yielding complex behaviors that depend on the mixing ratio. In particular, the P1-rich formulation (70/30) displays a *T*_gel_ at 27 °C with a *G*′ maximum at 32 °C, the P2-rich formulation (30/70) exhibits *T*_sol_ at 37 °C, while the 50/50 one demonstrates gel-like behavior in the entire temperature range, with a soft gel to strong gel transition between 20 and 30 °C. Three temperature regimes can be distinguished, regarding the *G*′ behavior, which reflects the degree of the network cross-linking (Figure 1a). At *T* < 20 °C (low *T* regime), the boronate ester dynamic covalent bonds act exclusively for the cross-linking of the sodium alginate chains. At *T* > 34 °C (high *T* regime), both cross-linking modes (hydrophobic and dynamic covalent debonding) coexist in the network, which weakens the *G*′ decrease compared to pure P2, owing to the progressive boronate ester bond disruption. Finally, the intermediate transition region (20–34 °C) is characterized by *G*′ augmentation due to the hydrophobic association of the P(NIPAM_86_-*co*-NtBAM_14_) grafted chains (thermothickening effect). However, this effect is less pronounced from that of pure P1 which is readily cross-linked by dynamic covalent bonding under the same conditions. Figure 1c presents *G*′ versus the blending ratios at 15 °C (low *T* regime) and 45 °C (high *T* regime). In the former case, *G*′ increases upon enriching the formulation with P2, while in the latter case, *G*′ values converge to the same value since the hydrophobic cross-linking acts almost exclusively as the boronate esters dissociate at higher temperatures, and especially at *T* > 37 °C. Among the blends, the most intense sol-to-gel transition is observed in the P1/P2 = 70/30 as the tangent (δ) is reduced by 442.1% upon heating from 20 to 37 °C, denoting the combined intramolecular interactions, i.e., boronate esters and hydrophobic binding. Likewise, the tan(δ) of P1/P2 = 50/50 is merely diminished by ca. 9.9% contrary to the gel-to-sol flux of P1/P2 = 70/30. At *T* > 37 °C, the boronate ester bonds dissociate as seen in P2, which tends to liquify and the systems are ruled by the hydrophobic interactions. Temperature-driven dissociation of boronate esters in the presence of thermoresponsive elements has also been observed in a previous study; it seems that this is more pronounced at elevated boronate content resulting in network disruption at higher temperatures. As a result, P1 and P1/P2 = 70/30 with low and moderate boronate ester content present optimum sol-to-gel transition at 24 and 27 °C, respectively.^23^ In addition, the complex viscosity of the sol-to-gel SIP (70/30) network is reinforced from 40.5 to 97.1 Pa s, i.e., a 140% increment indicating a promising bioprinting cell-protective carrier against extrusion for tissue regeneration applications (Figure 1d). The contribution of the boronate and hydrophobic associations tends to form a rigid (70/30) network as the average pore size diameter is decreased by ca. 69% compared to P1 at 37 °C and increased by ca. 15% compared to the P2 network at RT (Figure 1e–h). The samples for SEM analysis may have been slightly affected by the lyophilization process but probably without a significant influence on the porous texture due to the rapid freezing step.^24^

**Figure 2:**
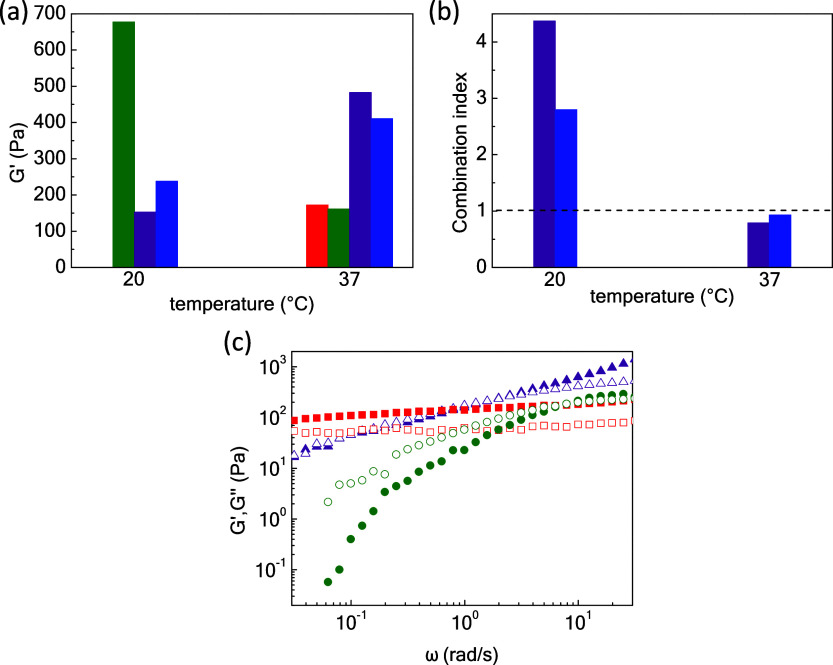
(a) Storage modulus of P1 (red), P1/P2 = 70/30 (purple), P1/P2 = 50/50 (blue), and P2 (olive); (b) combination index plot of the P1/P2 = 70/30 blend (purple) and P1/P2 = 50/50 (blue) as a result of storage modulus values from Figure 2a at 20 °C and 37 °C; (c) storage *G*′(closed) and loss *G*′’’ (open) moduli as a function of angular frequency of 5 wt % NaALG-*g*-P(NIPAM86-*co*-NtBAM14) (red, squares), NaALG-*g*-P(NIPAM86-*co*-NtBAM14)/NaALG-BA at a mixing ratio of 70/30 (wt %) (purple, triangles), and NaALG-BA (olive, circles) networks at 37 °C and a strain amplitude of 0.1%.

The storage moduli of the pristine networks, as well as the 70/30 and 50/50 blends, are depicted in Figure 2a. At room temperature, the 70/30 SIPN and the 50/50 blend present decreased storage moduli compared to *G*′ of P2 (*G*′ of ca. 677 Pa), i.e., reduced *G*′ of 77.5% for 70/30 (*G*′ of ca. 152 Pa) and 65% of 50/50 (*G*′ of ca. 238 Pa), respectively. The stronger 50/50 network at 20 °C is interpreted as being due to the higher boronate ester content compared to 70/30, which contributes to boronate ester formation. On the contrary, at physiological body temperature, the 70/30 SIPN presents 181% enhanced storage modulus (*G*′ of ca. 483 Pa), while the storage modulus of 50/50 is increased by 155% (*G*′ of ca. 411 Pa) compared to P1 (*G*′ of ca. 172 Pa). Upon heating at 37 °C, 70/30 is more potent compared to 50/50, as the first one is composed of 20 wt % more pristine P1 grafted with PNIPAM-based stickers adequate for hydrophobic association to build up the network. To better understand the mechanical properties of the networks, the synergistic effect of the two cross-linking mechanisms was estimated via the combination index (CI) at RT and 37 °C. Considering the blends as biocargo vehicles, emphasis is given to the SIP networks that present gel nature at physiological body temperature, i.e., P1/P2 = 70/30 and P1/P2 = 50/50. CΙ < 1 denotes synergism of the two mechanisms, CI = 1 represents additive enhancement, while CI > 1 displays antagonistic behavior of the cross-linkers (Table S1).^25−27^ In relation to the thermal profile of the network as depicted in Figure 2a, the CI of the 70/30 SIPN is 4.37, exhibiting an antagonistic effect for the blend network at 20 °C as the bulk P1 behaves as liquid-like at RT < *T*_gel_, surpassing the gel nature of P2. Surprisingly, upon heating, the CI becomes 0.79, reduced by ca. 82%, denoting the dominance of the hydrophobic cross-linking as boronate ester cross-links tend to dissociate at that temperature. Additionally, the CI of the 50/50 blend is 2.8 at RT, showing an antagonistic profile, and 0.93 at 37 °C, i.e., almost an additive effect of the cross-linkers. As a result, by simply mixing the same two alginate-based graft copolymers, the final materials react as totally different matrices at the molecular level. Considering bioapplications at physiological body temperature, the 70/30 blend presents enhanced abilities as SIPNs by 17.7% compared to the 50/50 matrix (Figure 2b).

To examine if the 70/30 blend could be used as a potential bioink, we studied the angular frequency dependence of the storage and loss moduli at a characteristic postinjectable/3D bioprintable temperature (37 °C) at a constant strain amplitude of 0.1%, in the linear viscoelastic regime (Figure 2c). At this temperature, the system behaves as a soft gel with a terminal relaxation time on the order of seconds. The underpinning network is characterized as semi-interpenetrating as the hydrophobic association of the thermoresponsive side chains is the predominant cross-linking mechanism. The storage modulus dominates the loss one, indicating the gel matrix formation at physiological body temperature at 6.28 rad/s, albeit the SIPN behaves as a viscous material after 3 s (*t*_rel_ = 1/ω_c_) as *G*′’ surpasses *G*′. This behavior is, in fact, characteristic of the gel-like profile of P1 as the storage and loss moduli are independent of the angular frequency due to the hydrophobic interactions and the sol-like P2 material at 37 °C due to the thermo-obedient boronate esters (Figure 2c).

**Figure 3:**
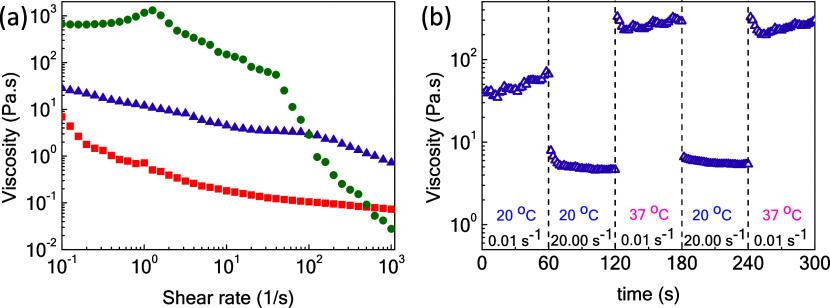
(a) Shear rate dependence of viscosity of 5 wt % P1 (red, squares), P1/P2 = 70/30 (purple, triangles), and P2 (olive, circles) hydrogels at a printing/injection temperature of 20 °C; (b) time dependence of viscosity of the 70/30 (wt %) blending system by altering the applied shear rate and temperature.

Concerning 3D printability and injectability for microtissue formation operations, the shear rate-dependent viscosity of the 70/30 SIPN and of the corresponding P1 and P2 constituents is evaluated at ambient conditions, i.e., at an extrusion temperature of 20 °C (Figure 3a). The viscosity of the P2 matrix is augmented by almost 2 orders of magnitude compared to the liquid-like P1, while the 70/30 SIPN exhibits intermediate comportment at RT. The linear viscoelastic regime of the solid-like P2 at room temperature at a low angular frequency of 6.28 rad/s is presented in Figure S7d, showing that the polymer matrix remains intact at up to 40% strain as storage and loss moduli are constant, implying that the sample is undisturbed. Importantly, all formulations exhibited shear-thinning behavior, which is the main requirement for injectability (printability) applications. Another important issue is the response of the formulation to sudden changes in temperature and shear. To address this in more detail, the influence of thermal and shear alterations upon the viscosity profile of the 70/30 blend was examined. Time sweep measurements were performed simulating an injection event by applying simultaneous stepwise changes of temperature (20 and 37 °C) and shear rate (0.01 and 20.00 s^–1^), as shown in Figure 3b. Initially, by altering the shear rate from 0.01 s^–1^ (e.g., preinjection) to 20.00 s^–1^ (e.g., actual injection event) at 20 °C, the viscosity of the hydrogel is reduced instantly by about 1 order of magnitude. At postinjection conditions, i.e., shear rate equal to 0.01 s^–1^ but at 37 °C, the viscosity of the network is enhanced promptly by 5000%, i.e., from 5 Pa s at injection thread to 250 Pa s at the postinjection one, due to the triggering of hydrophobic interactions of the PNIPAM-based side chains of the P1 fraction, as presented in Figure 3b. Therefore, the system exhibits excellent responsiveness to temperature and shear with only minor hysteresis upon shear/thermal fluctuations after a second cycle of alterations (4th and 5th steps), demonstrating injectability and self-healing capacity. In terms of injection comfort, the applied injection force *F* should be lower than 12 N.^28^ For instance, using a syringe with radius, *R*_s_ = 2.4 mm and a 27G needle (internal radius, *R*_n_ = 0.105 mm and length, *L* = 12.7 mm) and applying a flow rate of *Q*_v_ = 1 mL min^–1^, the apparent viscosity under the applied shear rate should be lower than 0.15 Pa s (see calculations in the Supporting Information). By extrapolating the shear-thinning data of Figure 3a, for the 70/30 formulation, we can fulfill this at shear rates of above 10^4^ s^–1^. Provided that the calculated shear rate, achieved under the above parameters (*Q*_v_ and *R*_s_), is about 18 × 10^3^ s^–1^ (see the Supporting Information), the 70/30 formulation can be considered comfortably acceptable to inject into humans.

**Figure 4:**
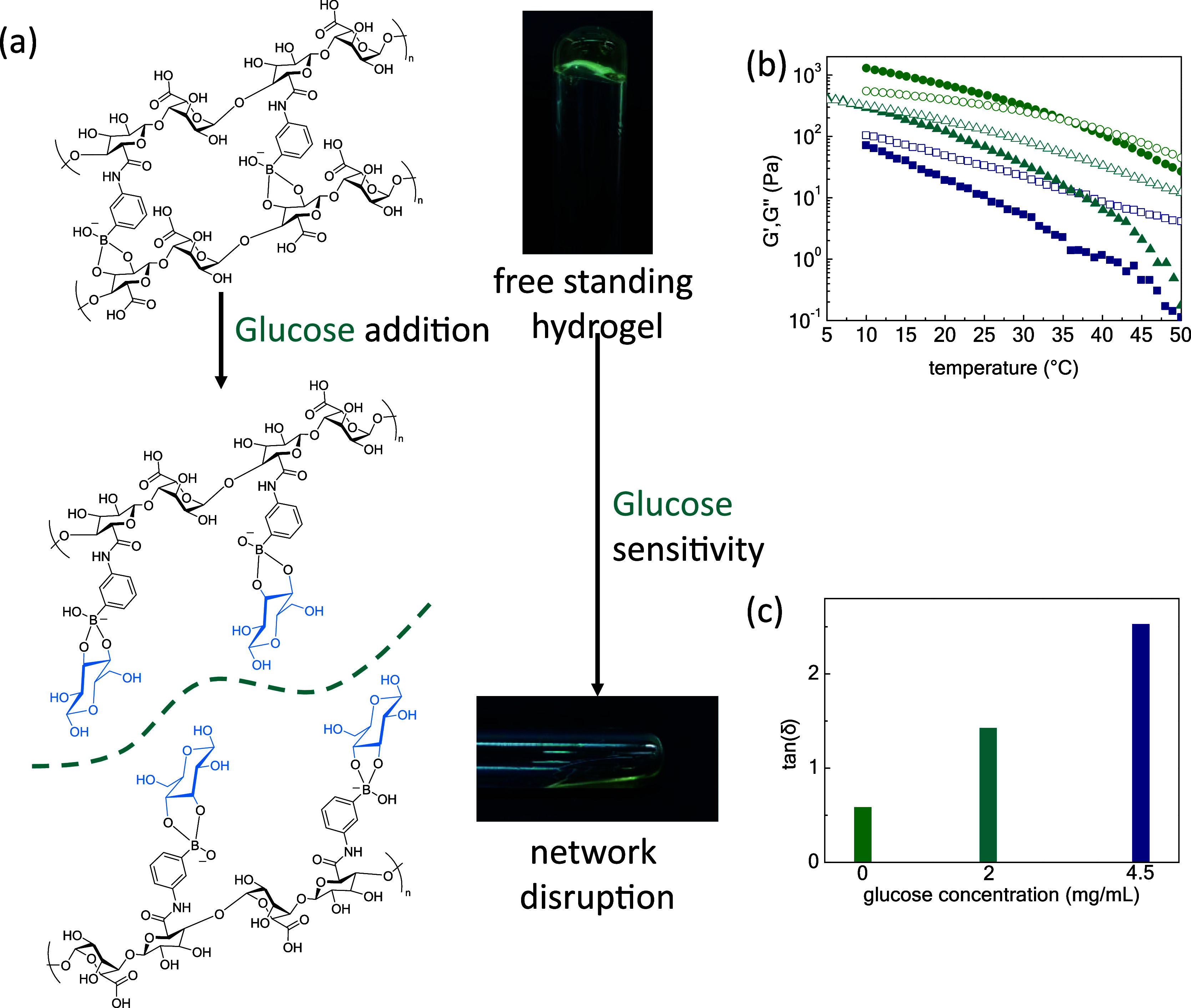
(a) Schematic representation of NaALG-BA network disruption after the addition of glucose; (b) *G*′ and *G*′’ as a function of temperature of 5 wt % NaALG-BA systems without (olive, circles) and with the addition of diabetes type I glucose level (cyan, triangles) and glucose concentration in Dulbecco’s modified Eagle’s medium (blue, squares) and (c) tan(δ) of 5 wt % NaALG-BA glucose-free and glycose-enriched gels at room temperature.

Next, the P2 polymer was examined as it exerts a dual function as an SIPN matrix augmentation block and as a glucose-responsive entity. It should be mentioned that the boronic acid stickers are prone to pH alterations triggering network formation at pH levels above their p*K*_a_ value (ca. 8.8). At physiological pH, the ionized boronate tetrahedral esters are ca. 5%, which is still a sufficient amount to promote gelation.^29−31^ The pH threshold of the P2 gelation was further explored above 7.1 due to the pH-sensitive boronic moieties upon heating, as already seen in Figure S5. Especially, the pH increase to 9 leads to stronger gel formation as the storage modulus surpasses the loss one during the whole thermal range. The tangent delta of the P2 at physiological pH is 0.58, i.e., 9.6 times more compared with the tan delta value of 0.06 at pH 9 and at RT, as the amount of charged boronate esters is enhanced. Furthermore, as temperature hampers gel formation, at physiological body temperature the tangent delta at alkaline pH is 0.08, retaining the gel nature, i.e., ca. 12% decreased compared to the ca. 1.04 value of the tan delta at pH 7.4 (Figure S6). Consequently, glucose addition should challenge the integrity of the boronate ester cross-linking mechanism among alginate chains as the diol groups of the monosaccharide could induce progressive network disruption by additional competition reactions (Figure 4a).^32−35^ At RT and physiological body pH conditions, the network is dominated by the boronate esters; however, the addition of glucose at disease-related levels (i.e., diabetes type I glucose level of 2 mg/mL) prompts polymer-bound ester bond dissociation. At 10 °C, i.e., a temperature that does not affect the integrity of boronate esters, the 5 wt % aqueous network is conducted by formed boronate esters, which represent 22.6 wt % of the NaALG-*g*-BA copolymer. Concerning the antagonistic mechanism of glucose over the network due to the BA/glucose esters, 16.9% of boronic/glucose blocks are accomplished by calculating the molar concentration of glucose [Glu] over the molar concentration of boronate acid moieties [BA]. Further addition of glucose up to 4.5 mg/mL, i.e., the concentration of high glucose DMEM, leads to rapid liquification as this amount of sugar interacts with the [Glu]/[BA] = 38.4% of the existing boronic acid components (Figure 4b). Notably, the preponderance of the elastic or viscous behavior is indicated by tangent delta, which is equal to 0.6 of the P2, which indicates the solid profile of the material. The existence of glucose at diabetes type I level 2 mg/mL contributes to the disorganization of the 3D matrix as tangent delta is ca. 144% increased compared to the tan(δ) of P2. Further addition of glucose to the DMEM level induces even more extensive dismantling of polymer–polymer boronate esters, as tan(δ) becomes 2.5, that is, 332% increase (Figure 4c).

Additionally, the boronic acid-enriched SIPN readily forms boronate esters with 1,2- and 1,3-diols of saccharides found on the alginate backbone and the glycocalyx of mammalian cells.^36−39^ Here, the boronic acid pendants are shown to promote cell-polymer matrix associations acting as cellular glues that induce the formation of cell aggregates.

**Figure 5:**
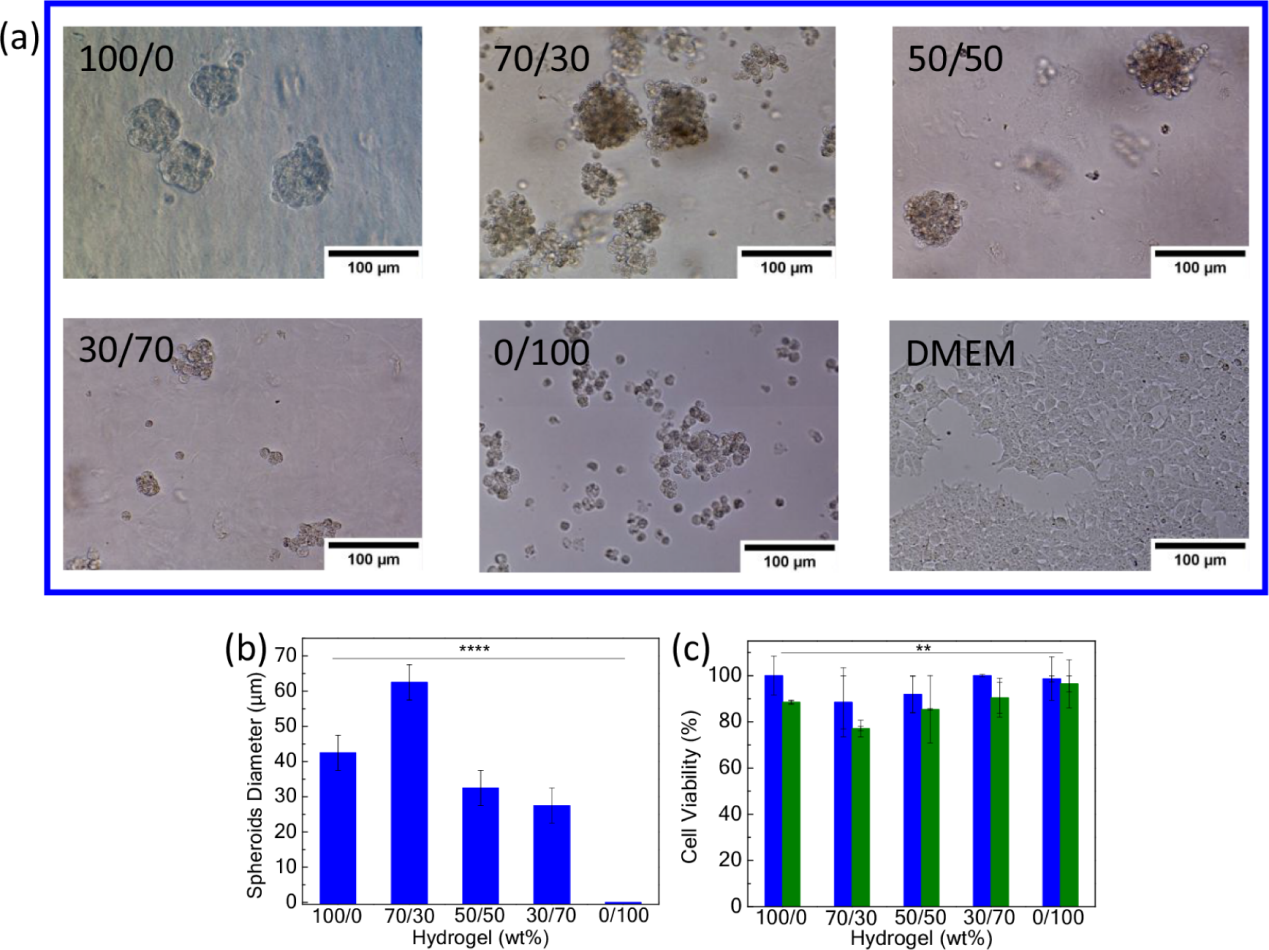
(a) Cell aggregation formation into the various 5 wt % P1/P2 hydrogel blends after 24 h; (b) cell spheroid diameter growth after 24 h and (c) HEK293T cell viability after 24 h (blue) and 48 h (olive).

Hence, we focused on the precise conditions needed to rapidly form cell spheroids with the use of 5 wt % P1 and P2 blends. Human embryonic kidney 293T (HEK293T) cells were mixed with various polymer hydrogels and examined for their capability to form cell aggregates (Figure 5a). First, 20 × 10^3^ HEK293T cells per well (48-well plate) were mixed with P1 and were found to assemble into spheroids due to the well-formed P1 gel at 37 °C > *T*_gel_ = 24 °C (pH 7.4, 24 h), even though this polymer lacks specific cell-surface targeting properties. We presume that the polymer acts as an intermediate hydrophobic bridge which, in combination with the formation of a more robust 3D matrix, leads to the formation of cell spheroids. On the other hand, moderate cell aggregate formation was observed when cells were mixed solely with the boronate-rich P2, as the polymer is in liquid form under the same conditions. The optimum 5 wt % P1/P2 SIPN ratio to form cell spheroids rapidly was found to be 70/30 wt %. At this blend ratio, P1 reinforces the 3D network, while P2 promotes extensive cell membrane interactions via boronate ester bridging with the cells’ glycocalyx. Next, we studied the role of the blend ratio in the mean cell spheroid diameter. It was found that the P1/P2 = 70/30 blend formed spheroids of diameter 62.5 μm, ca. 92% increase compared to the P1/P2 = 50/50 blend (mean diameter, 32.5 μm), ca. 127% increase compared to the P1/P2 = 30/70 blend (mean diameter, 27.5 μm), and ca. 47% increase compared to P1 alone (mean diameter, 42.5 μm) (Figure 5b). In addition, all SIPNs exhibited excellent cell viability levels above 77% after 48 h (Figure 5c). Furthermore, the rheological behavior of the optimum 70/30 network for facile cell spheroid formation was evaluated. Three replicates of the gel were studied at a fixed temperature range (Figure S4) showing adequate reproducibility of the network formation across the tested samples.

**Figure 6:**
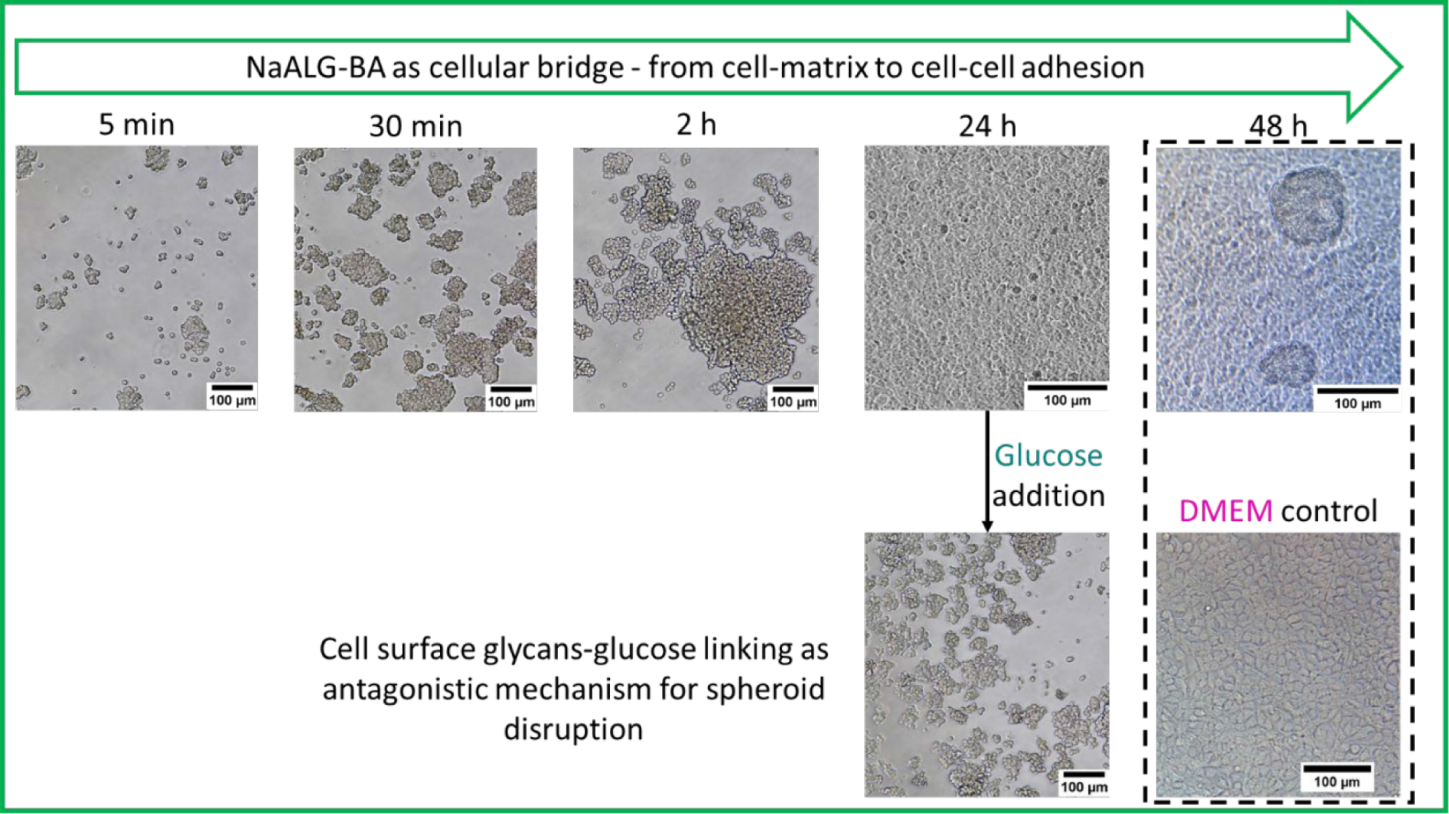
Optical microscopic images of HEK 293T cells in 25 × 10^–4^ wt % P2 gel (below gelation point) at different time points up to 48 h observing the rapid cell–cell adhesive conformations which are disordered by glucose addition; HEK293T embedded in DMEM were used as the control sample.

Since P2 is responsible for specific ligand–receptor interactions and is likely the key element for spheroid formation, we conducted additional experiments to probe its role at dilute concentrations well below the gelation point (25 × 10^–4^ wt %, while the actual minimum concentration required to form a polymer network is more than 4 wt %, Figure S7). The presence of the boronic acid, even at minute concentrations, was sufficient to induce cell aggregation in less than 30 min owing to the rapid interaction with the cell surface diols. During a 48-h proliferation period, the cells were found to form progressively larger cell aggregates due to the gradual boronate ester formation on the cell membrane, a pattern that was not seen in the DMEM control (that is, in the absence of any polymer) (Figure 6). The specificity of the interaction could be probed by dissociation experiments by the addition of 100 mM glucose, which induced aggregate disassembly.

Furthermore, the addition of excess glucose (i.e., 10× more than diabetic glucose concentration or 4× more than DMEM glucose content) led to cell aggregate dissociation owing to the antagonistic coupling of boronic acids with free glucose instead of cell-bound diols (Figure 6).

**Figure 7:**
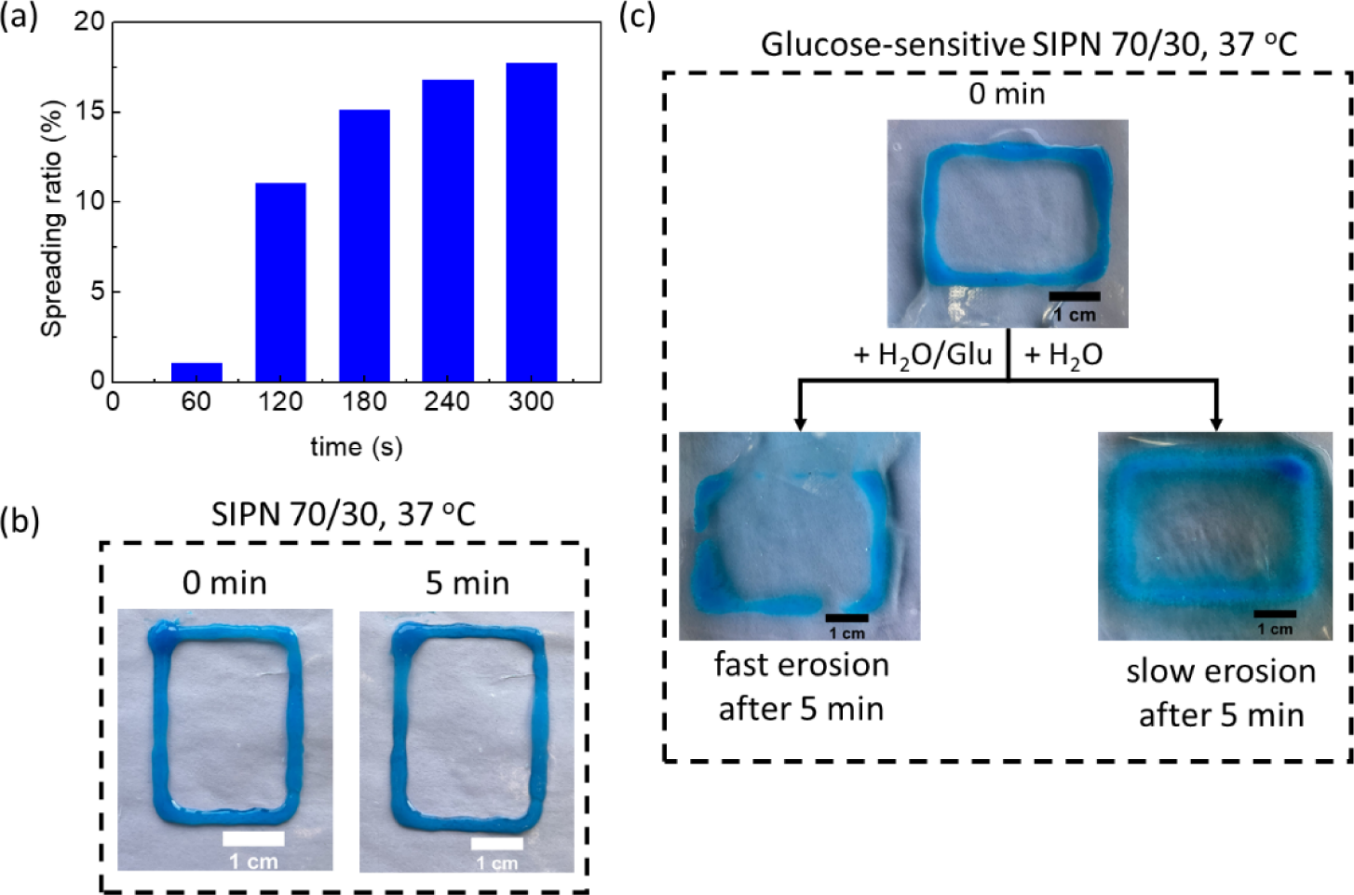
(a) Spreading ratio (%) as calculated from the line width of the 3D-printed 5 wt % blend domains at P1/P2 = 70/30 divided by the 25G needle inner diameter and macroscopic images of the scaffolds described above at 37 °C. (b) Optical illustration of the spreading ratio of the previously mentioned blend after 5 min at 37 °C. (c) Glucose-responsive disruption of the P1/P2 = 70/30 blend after moisturizing the samples with solely aqueous or glucose-rich solutions.

Having established that the P1/P2 = 70/30 blend is the optimum formulation for 3D network and cell spheroid formation, we assessed its 3D printability as a bioink under mild conditions, i.e., its biocompatibility and injectability.^40,41^ First, the 5 wt % SIPN remained in a 5 mL Luer lock syringe at room temperature and was 3D printed directly onto a heated bed at 37 °C by extrusion through a 25-G needle. The hydrogel was readily printed to a smooth result. To pursue more precise patterns, the height of each layer was defined at 0.2 mm, and all 3D patterns were printed with five layers with final dimensions of 2 × 2.5 × 0.1 cm^3^. The accuracy of each print was evaluated by the spreading ratio, which is defined as the line width divided by the needle inner diameter, i.e., 0.26 mm. At these conditions, the precision of the soft prints was retained due to the hydrophobic self-associations of P1 at *T* > *T*_gel_ of the blend, deviating only ca. 17.7% after five minutes, as shown in Figure 7a,b. The printed blend was tested at body temperature by moisturizing it with glucose-enriched and glucose-free aqueous solutions to fully probe the role of boronate ester dissociation on network disintegration. The glucose-free hydrated sample showed significantly slower erosion due to the persistence of the hydrophobic associations of P1. However, glucose addition led to rapid pattern dismantling of the SIPN within 5 min, clearly demonstrating the role of P2 in the structural integrity of the polymer matrix (Figure 7c).

**Figure 8:**
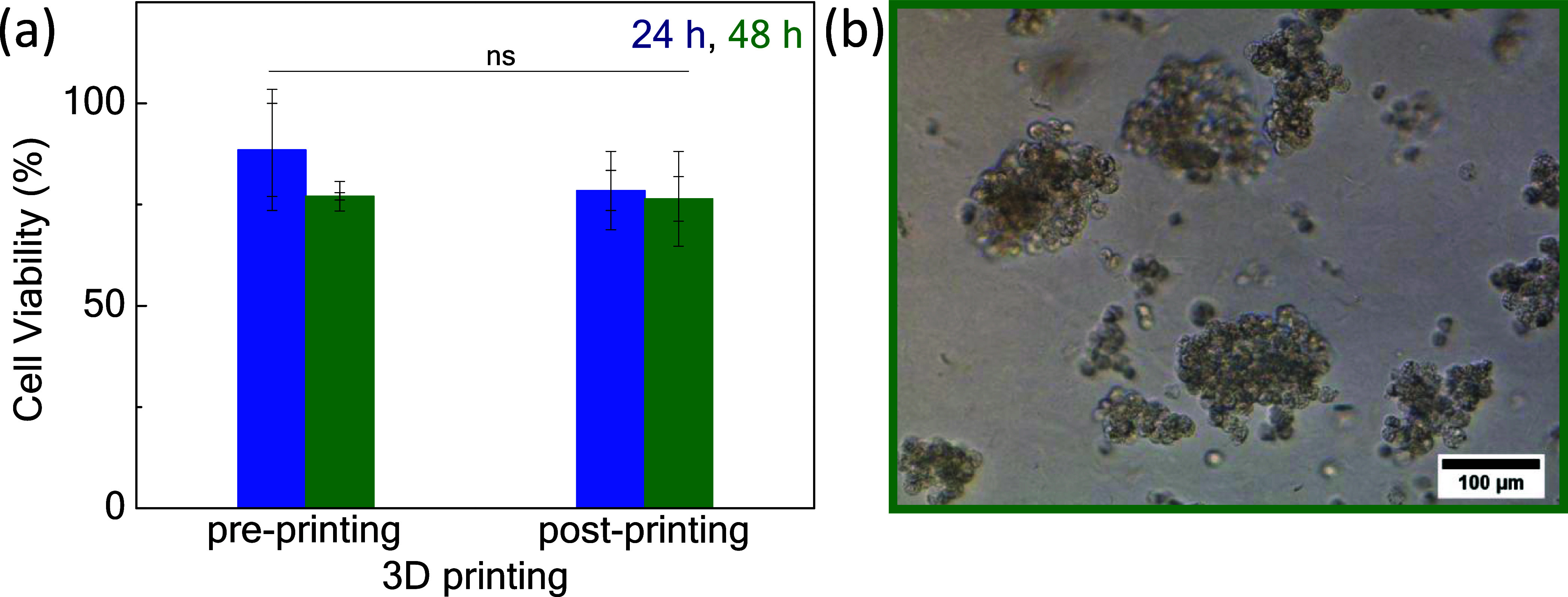
(a) Cell viability of HEK 293T cells in 70/30 hydrogel matrices pre/post printing after 24 h (blue) and 48 h (olive); (b) post printing cell aggregate formation within the P1/P2 = 70/30 hydrogel after 24 h.

Finally, HEK293T cells were embedded in a 5 wt % 70/30 (in DMEM) hydrogel at a ratio of 20 × 10^3^ cells per 200 μL gel in order to evaluate the bioprinting process in terms of cell viability. The cell-rich hydrogels were loaded into a 5 mL syringe of the 3D printer at room temperature, followed by printing arbitrary structures in 10 cm cell culture plates; the cell viability of the extruded cells was measured at 24 and 48 h under cell culture conditions. Cell viability reached ca. 78%, i.e., decreased by 10% compared with the nonprinted cells after 24 h, presumably due to the applied shear stress. At 48 h, the cell viability was virtually unchanged compared to the nonprinted cells (ca. 76%, Figure 8a. At room temperature, the sol 70/30 seems to protect cells by its facile printing; albeit at 37 °C, similar behavior with nonprinted 70/30 cell carrier is observed after 24 h. As expected, the 70/30 blend could induce the formation of cell aggregates 24 h after the printing process (Figure 8b. We attribute this property to the hydrophobic association of the PNIPAM-based P1 chains at 37 °C that partly sustains a 3D microenvironment while the boronate esters of P2 promote the formation of aggregates via cell–membrane interactions.

## 4 Conclusions

In summary, we demonstrated the development of a 3D SIPN bioink based on sodium alginate copolymers modified with thermoresponsive stickers and boronic acids. It was shown that the mechanical properties of the resulting hydrogels can be finely tuned by altering the weight percentage of the two graft copolymers in the blend, resulting in materials with significant functional variability. The SIPNs promote in situ cell aggregation, which can be regulated by thermal stimulation or the addition of glucose. A systematic investigation of the gelation conditions identified an optimal copolymer blend—70/30 wt % ratio of NaALG-*g*-P(NIPAM-*co*-NtBAM) to NaALG-*g*-BA—that balances robust polymer network formation, efficient cell encapsulation, and effective cell spheroid formation, while also maintaining good injectability and printability. The two copolymers are relatively easy to synthesize, and the simple mixing protocols allow for the creation of printable matrices with tunable properties, suitable for cell encapsulation strategies. This approach holds promise for broader applications, such as microtissue engineering, 3D matrices for drug screening, and injectable biomaterials for cell delivery in biotherapeutics.

## Supporting Information Available

^1^H NMR and FTIR spectra, additional rheological data, experimental details of the combination index, and injectability calculations (PDF)

## Abbreviations

SIPN: semi-interpenetrated polymer network
RT: room temperature
P1: NaALG-g-P(NIPAM-co-NtBAM)
P2: NaALG-g-BA
HEK293T: human embryonic kidney cell line
DMEM: Dulbecco’s modified Eagle’s medium

## References

[ref1] Yamazawa Y.; Kato H.; Nakaji-Hirabayashi T.; Yoshikawa C.; Kitano H.; Ohno K.; Saruwatari Y.; Matsuoka K. Bioinactive Semi-Interpenetrating Network Gel Layers: Zwitterionic Polymer Chains Incorporated in a Cross-Linked Polymer Brush. J. Mater. Chem. B 2019, 7 (27), 4280–4291. 10.1039/C8TB03228A.

[ref2] Tuan H. N.; Nhu V. T. Synthesis and Properties of PH-Thermo Dual Responsive Semi-IPN Hydrogels Based on N,N’-Diethylacrylamide and Itaconamic Acid. Polymers 2020, 12, 1139 10.3390/polym12051139. 32429371 PMC7285170

[ref3] Rwei S.-P.; Tuan H. N. A.; Chiang W.-Y.; Way T.-F. Synthesis and Characterization of PH and Thermo Dual-Responsive Hydrogels with a Semi-IPN Structure Based on N-Isopropylacrylamide and Itaconamic Acid. Materials 2018, 11 (5), 696 10.3390/ma11050696. 29710793 PMC5978073

[ref4] Radhakrishnan J.; Subramanian A.; Sethuraman S. Injectable Glycosaminoglycan–Protein Nano-Complex in Semi-Interpenetrating Networks: A Biphasic Hydrogel for Hyaline Cartilage Regeneration. Carbohydr. Polym. 2017, 175, 63–74. 10.1016/j.carbpol.2017.07.063. 28917911

[ref5] Rinoldi C.; Lanzi M.; Fiorelli R.; Nakielski P.; Zembrzycki K.; Kowalewski T.; Urbanek O.; Grippo V.; Jezierska-Woźniak K.; Maksymowicz W.; Camposeo A.; Bilewicz R.; Pisignano D.; Sanai N.; Pierini F. Three-Dimensional Printable Conductive Semi-Interpenetrating Polymer Network Hydrogel for Neural Tissue Applications. Biomacromolecules 2021, 22 (7), 3084–3098. 10.1021/acs.biomac.1c00524. 34151565 PMC8462755

[ref6] Alemán J. V.; Chadwick A. V.; He J.; Hess M.; Horie K.; Jones R. G.; Kratochvíl P.; Meisel I.; Mita I.; Moad G.; et al. Definitions of Terms Relating to the Structure and Processing of Sols, Gels, Networks, and Inorganic-Organic Hybrid Materials (IUPAC Recommendations 2007). Pure Appl.Chem. 2007, 79 (10), 1801–1829. 10.1351/pac200779101801.

[ref7] Dvir-Ginzberg M.; Gamlieli-Bonshtein I.; Agbaria R.; Cohen S. Liver Tissue Engineering within Alginate Scaffolds: Effects of Cell-Seeding Density on Hepatocyte Viability, Morphology, and Function. Tissue Eng. 2003, 9 (4), 757–766. 10.1089/107632703768247430. 13678452

[ref8] Li Y.; Kumacheva E. Hydrogel Microenvironments for Cancer Spheroid Growth and Drug Screening. Sci. Adv. 2024, 4 (4), eaas8998 10.1126/sciadv.aas8998. PMC5922799 29719868

[ref9] Ioannidis K.; Danalatos R. I.; Champeris Tsaniras S.; Kaplani K.; Lokka G.; Kanellou A.; Papachristou D. J.; Bokias G.; Lygerou Z.; Taraviras S. A Custom Ultra-Low-Cost 3D Bioprinter Supports Cell Growth and Differentiation. Front. Bioeng. Biotechnol. 2020, 8, 580889 10.3389/fbioe.2020.580889. 33251196 PMC7676439

[ref10] Amaral A. J. R.; Pasparakis G. Macromolecular Cell Surface Engineering for Accelerated and Reversible Cellular Aggregation. Chem. Commun. 2015, 51 (99), 17556 10.1039/C5CC07001E. 26478926

[ref11] Decarli M. C.; Amaral R.; Dos Santos D. P.; Tofani L. B.; Katayama E.; Rezende R. A.; da Silva J. V.; Swiech K.; Suazo C. A.; Mota C.; et al. Cell Spheroids as a Versatile Research Platform: Formation Mechanisms, High Throughput Production, Characterization and Applications. Biofabrication 2021, 13 (3), 032002 10.1088/1758-5090/abe6f2. 33592595

[ref12] Amaral A. J. R. A. J. R.; Pasparakis G. Cell Membrane Engineering with Synthetic Materials: Applications in Cell Spheroids, Cellular Glues and Microtissue Formation. Acta Biomater. 2019, 90, 21 10.1016/j.actbio.2019.04.013. 30986529

[ref13] Amaral A. J. R.; Pasparakis G. Rapid Formation of Cell Aggregates and Spheroids Induced by a “Smart” Boronic Acid Copolymer. ACS Appl. Mater. Interfaces 2016, 8 (35), 22930–22941. 10.1021/acsami.6b07911. 27571512

[ref14] Ioannidis K.; Angelopoulos I.; Gakis G.; Karantzelis N.; Spyroulias G. A.; Lygerou Z.; Taraviras S. 3D Reconstitution of the Neural Stem Cell Niche: Connecting the Dots. Front. Bioeng. Biotechnol. 2021, 9, 705470 10.3389/fbioe.2021.705470. 34778223 PMC8581349

[ref15] Safakas K.; Saravanou S.-F.; Iatridi Z.; Tsitsilianis C. Alginate-g-PNIPAM-Based Thermo/Shear-Responsive Injectable Hydrogels: Tailoring the Rheological Properties by Adjusting the LCST of the Grafting Chains. Int. J. Mol. Sci. 2021, 22 (8), 3824 10.3390/ijms22083824. 33917134 PMC8067843

[ref16] Iatridi Z.; Saravanou S.-F.; Tsitsilianis C. Injectable Self-Assembling Hydrogel from Alginate Grafted by P(N-Isopropylacrylamide-Co-N-Tert-Butylacrylamide) Random Copolymers. Carbohydr. Polym. 2019, 219, 344–352. 10.1016/j.carbpol.2019.05.045. 31151534

[ref17] Saravanou S. F.; Ioannidis K.; Dimopoulos A.; Paxinou A.; Kounelaki F.; Varsami S. M.; Tsitsilianis C.; Papantoniou I.; Pasparakis G. Dually Crosslinked Injectable Alginate-Based Graft Copolymer Thermoresponsive Hydrogels as 3D Printing Bioinks for Cell Spheroid Growth and Release. Carbohydr. Polym. 2023, 312, 120790 10.1016/j.carbpol.2023.120790. 37059530

[ref18] Hong S. H.; Kim S.; Park J. P.; Shin M.; Kim K.; Ryu J. H.; Lee H. Dynamic Bonds between Boronic Acid and Alginate: Hydrogels with Stretchable, Self-Healing, Stimuli-Responsive, Remoldable, and Adhesive Properties. Biomacromolecules 2018, 19 (6), 2053–2061. 10.1021/acs.biomac.8b00144. 29601721

[ref19] Amaral A. J. R.; Pasparakis G. Rapid Formation of Cell Aggregates and Spheroids Induced by a “Smart” Boronic Acid Copolymer. ACS Appl. Mater. Interfaces 2016, 8 (35), 22930 10.1021/acsami.6b07911. 27571512

[ref20] Stathopoulou A.; Roukos V.; Petropoulou C.; Kotsantis P.; Karantzelis N.; Nishitani H.; Lygerou Z.; Taraviras S. Cdt1 Is Differentially Targeted for Degradation by Anticancer Chemotherapeutic Drugs. PLoS One 2012, 7 (3), e34621 10.1371/journal.pone.0034621. 22479651 PMC3316709

[ref21] Samokhin A. S. Syringe Pump Created Using 3D Printing Technology and Arduino Platform. J. Anal. Chem. 2020, 75 (3), 416–421. 10.1134/S1061934820030156.

[ref22] Guo H.; de Magalhaes Goncalves M.; Ducouret G.; Hourdet D. Cold and Hot Gelling of Alginate-Graft-PNIPAM: A Schizophrenic Behavior Induced by Potassium Salts. Biomacromolecules 2018, 19 (2), 576–587. 10.1021/acs.biomac.7b01667. 29284259

[ref23] Amaral A. J. R.; Emamzadeh M.; Pasparakis G. Transiently Malleable Multi-Healable Hydrogel Nanocomposites Based on Responsive Boronic Acid Copolymers. Polym. Chem. 2018, 9 (4), 525–537. 10.1039/c7py01202k.

[ref24] Efthymiou C.; Williams M. A. K.; McGrath K. M. Revealing the Structure of High-Water Content Biopolymer Networks: Diminishing Freezing Artefacts in Cryo-SEM Images. Food Hydrocolloids 2017, 73, 203–212. 10.1016/j.foodhyd.2017.06.040.

[ref25] Chou T.-C. Drug Combination Studies and Their Synergy Quantification Using the Chou-Talalay Method. Cancer Res. 2010, 70 (2), 440–446. 10.1158/0008-5472.CAN-09-1947. 20068163

[ref26] Barnett C.; Joubert F.; Iliopoulou A.; Álvarez R. S.; Pasparakis G. Photochemical Internalization Using Natural Anticancer Drugs, Antimetabolites, and Nanoformulations: A Systematic Study against Breast and Pancreatic Cancer Cell Lines. Mol. Pharmaceutics 2023, 20 (3), 1818–1841. 10.1021/acs.molpharmaceut.2c01012. 36802639

[ref27] Foucquier J.; Guedj M. Analysis of Drug Combinations: Current Methodological Landscape. Pharmacol. Res. Perspect. 2015, 3 (3), e00149 10.1002/prp2.149. 26171228 PMC4492765

[ref28] Robinson T. E.; Hughes E. A. B.; Bose A.; Cornish E. A.; Teo J. Y.; Eisenstein N. M.; Grover L. M.; Cox S. C. Filling the Gap: A Correlation between Objective and Subjective Measures of Injectability. Adv. Healthcare Mater. 2020, 9 (5), 1901521 10.1002/adhm.201901521. 31977153

[ref29] Pettignano A.; Grijalvo S.; Häring M.; Eritja R.; Tanchoux N.; Quignard F.; Díaz Díaz D. Boronic Acid-Modified Alginate Enables Direct Formation of Injectable, Self-Healing and Multistimuli-Responsive Hydrogels. Chem. Commun. 2017, 53 (23), 3350–3353. 10.1039/C7CC00765E. 28261723

[ref30] Chtchigrovsky M.; Lin Y.; Ouchaou K.; Chaumontet M.; Robitzer M.; Quignard F.; Taran F. Dramatic Effect of the Gelling Cation on the Catalytic Performances of Alginate-Supported Palladium Nanoparticles for the Suzuki–Miyaura Reaction. Chem. Mater. 2012, 24 (8), 1505–1510. 10.1021/cm3003595.

[ref31] Gosecki M.; Gosecka M. Boronic Acid Esters and Anhydrates as Dynamic Cross-Links in Vitrimers. Polymers 2022, 14 (4), 842 10.3390/polym14040842. 35215755 PMC8962972

[ref32] Gaballa H.; Theato P. Glucose-Responsive Polymeric Micelles via Boronic Acid–Diol Complexation for Insulin Delivery at Neutral PH. Biomacromolecules 2019, 20 (2), 871–881. 10.1021/acs.biomac.8b01508. 30608155

[ref33] Hong S. H.; Shin M.; Park E.; Ryu J. H.; Burdick J. A.; Lee H. Alginate-Boronic Acid: PH-Triggered Bioinspired Glue for Hydrogel Assembly. Adv. Funct. Mater. 2020, 30 (26), 1908497 10.1002/adfm.201908497.

[ref34] Dong Y.; Wang W.; Veiseh O.; Appel E. A.; Xue K.; Webber M. J.; Tang B. C.; Yang X.-W.; Weir G. C.; Langer R.; Anderson D. G. Injectable and Glucose-Responsive Hydrogels Based on Boronic Acid–Glucose Complexation. Langmuir 2016, 32 (34), 8743–8747. 10.1021/acs.langmuir.5b04755. 27455412 PMC5242094

[ref35] Yao Y.; Zhao L.; Yang J.; Yang J. Glucose-Responsive Vehicles Containing Phenylborate Ester for Controlled Insulin Release at Neutral PH. Biomacromolecules 2012, 13 (6), 1837–1844. 10.1021/bm3003286. 22537190

[ref36] Ellis G. A.; Palte M. J.; Raines R. T. Boronate-Mediated Biologic Delivery. J. Am. Chem. Soc. 2012, 134 (8), 3631–3634. 10.1021/ja210719s. 22303837 PMC3304437

[ref37] Karimi F.; Collins J.; Heath D. E.; Connal L. A. Dynamic Covalent Hydrogels for Triggered Cell Capture and Release. Bioconjugate Chem. 2017, 28 (9), 2235–2240. 10.1021/acs.bioconjchem.7b00360. 28809538

[ref38] Tan Y.; Wu J.; Song L.; Zhang M.; Hipolito C. J.; Wu C.; Wang S.; Zhang Y.; Yin Y. Merging the Versatile Functionalities of Boronic Acid with Peptides. Int. J. Mol. Sci. 2021, 22 (23), 12958 10.3390/ijms222312958. 34884766 PMC8657650

[ref39] António J. P. M.; Russo R.; Carvalho C. P.; Cal P. M. S. D.; Gois P. M. P. Boronic Acids as Building Blocks for the Construction of Therapeutically Useful Bioconjugates. Chem. Soc. Rev. 2019, 48 (13), 3513–3536. 10.1039/C9CS00184K. 31157810

[ref40] Tarassoli S. P.; Jessop Z. M.; Jovic T.; Hawkins K.; Whitaker I. S. Candidate Bioinks for Extrusion 3D Bioprinting—A Systematic Review of the Literature. Front. Bioeng. Biotechnol. 2021, 9, 616753 10.3389/fbioe.2021.616753. 34722473 PMC8548422

[ref41] Piras C. C.; Smith D. K. Multicomponent Polysaccharide Alginate-Based Bioinks. J. Mater. Chem. B 2020, 8 (36), 8171–8188. 10.1039/D0TB01005G. 32776063

