# Supplementary material for: Injectable and 3D-Printable Semi-Interpenetrating Polymer Networks Based on Modified Sodium Alginate for Cell Spheroid Formation
Source: Biomacromolecules. 2024 Dec 30;26(1):567–78. doi: 10.1021/acs.biomac.4c01343 (PMC11733927; doi:10.1021/acs.biomac.4c01343)
Supplement: Supplementary file 1 — bm4c01343_si_001.pdf [file bm4c01343_si_001.pdf]

Supporting Information

Injectable and 3D-Printable Semi-Interpenetrating Polymer Networks Based on Modified Sodium Alginate for Cell Spheroid Formation

Sofia Falia Saravanou[1], Thomai Samouilidou[2], Constantinos Tsitsilianis[1], Stavros Taraviras[2] and George Pasparakis[1]*

[1]Department of Chemical Engineering, University of Patras, Greece

[2]Department of Physiology, School of Medicine, University of Patras, Greece

^{1}H NMR

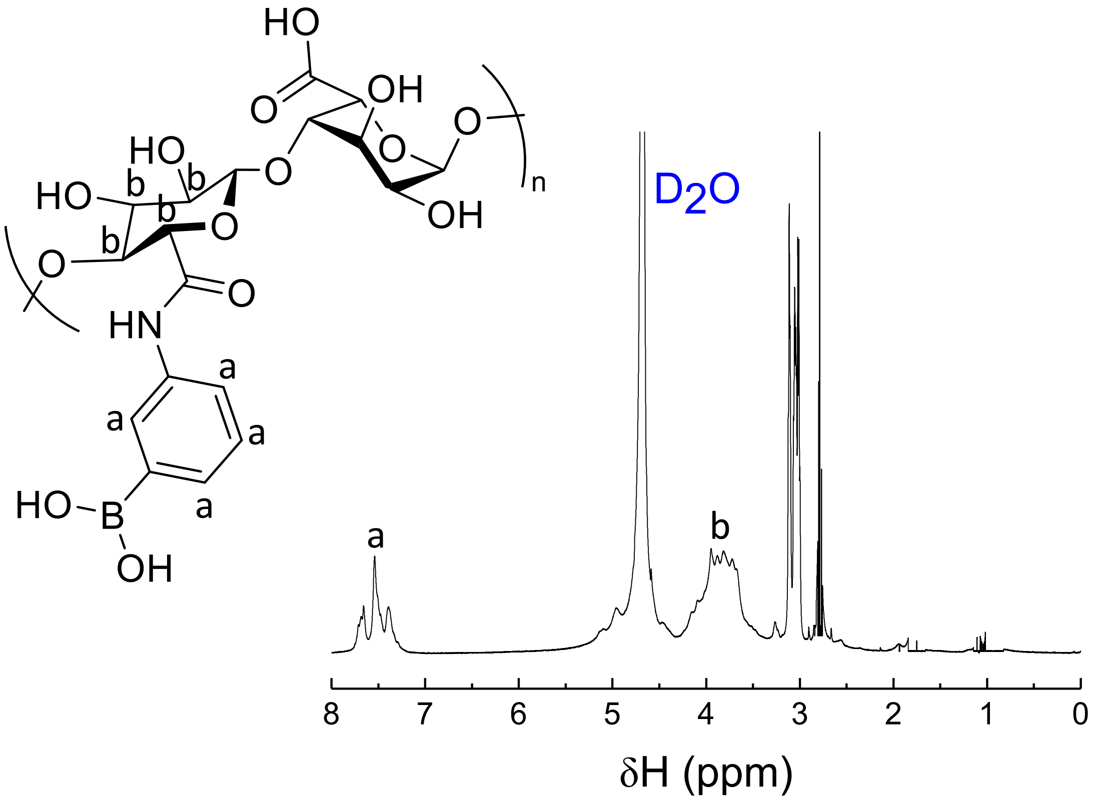

Figure S1. ^{1}H NMR spectra of NaALG-g-BA in D_2O.

FTIR

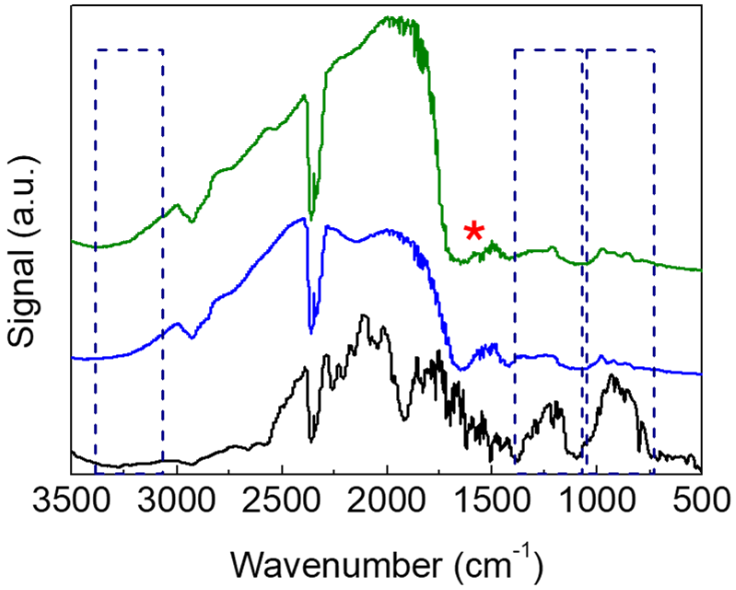

Figure S2. FTIR spectra of NaALG-g-BA (olive), NaALG (blue), and 3-aminophenyl boronic acid (black).

Calculation of the combination index

The combinational effect of the hydrophobic and boronic esters crosslinking interactions was investigated through the combination index, that represents antagonistic (CI>1), additive (CI=1) or synergistic contribution (CI<1).[1,2] The CI value is calculated from formula (1):

$$\boldsymbol{CI = \frac{G'_{P1}+G'_{P2}}{G'_{P1/P2}}} \quad \textbf{(1)}$$

where, G_{P1}', $G_{P2}'$are the storage modulus values of the crosslinked NaALG-g-P(NIPAM-co-NtBAM) and NaALG-g-BA networks respectively and $G_{P1/p2}'$ is the elastic modulus of the hybrid networks. The CI values at RT and 37 °C are presented in Table S1.

Table S1. Combination index at room and physiological body temperature of the blends.

P1/P2	CI (20 °C)	CI (37 °C)
70/30	4.37	2.80
50/50	0.79	0.93

Glucose treatment of HEK293T cells

24 h proliferated HEK 293T cells embedded below the gelation of the NaALG-g-BA/DMEM solution at a concentration of 25×10^{-4} wt. % were treated with 0.1 mM glucose/DMEM solution. First 50 μL glucose solution were added in the 48-well plates every five minutes. No difference was observed after 15 minutes as the cell population was very dense considering that NaALG-g-BA promoted esters with diols on the cellular surface accelerating the formation of cell-cell aggregations. 500 μL of 0.1 mM glucose were added gradually and the plate was placed in the incubator. After 90 minutes (i.e., required time to allow for full glucose diffusion across the whole volume of each 48-well), dissociation of the cellular layers was observed due to the antagonistic mechanism of glucose with the NaALG-g-BA/cells esters.

Rheological Results

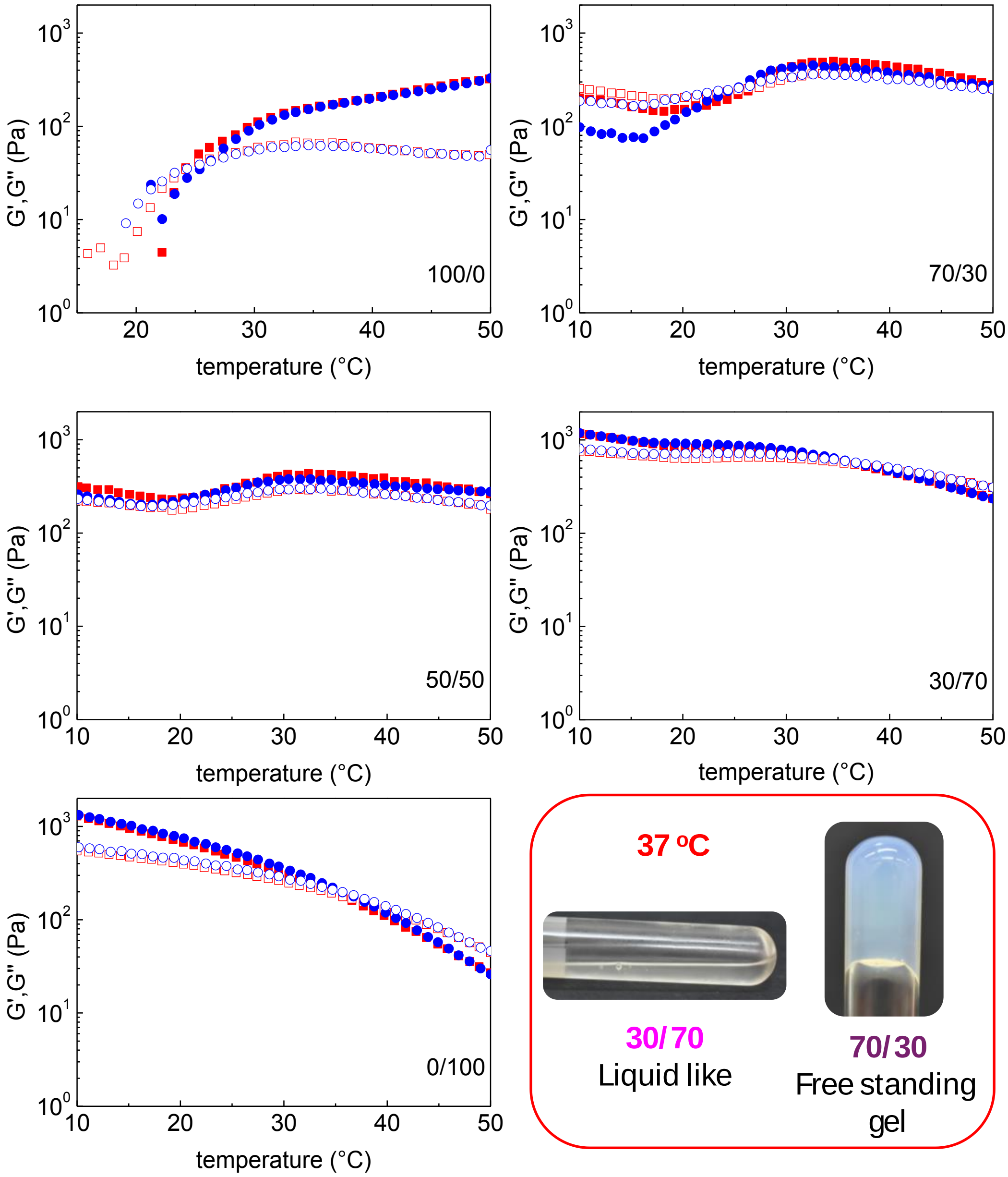

Figure S3. Storage modulus (closed) and loss modulus (open symbols) as a function of temperature of 5 wt.% P1/P2 SIPN hydrogels at frequency 1 Hz, strain amplitude 0.1% and during heating (red) and cooling cycle (blue) with heating rate of 1 °C/min; Image of 5 wt.% P1/P2 30/70 and 70/30 blends at physiological body temperature with the first one being in the sol-like phase and the second one as gel-like material.

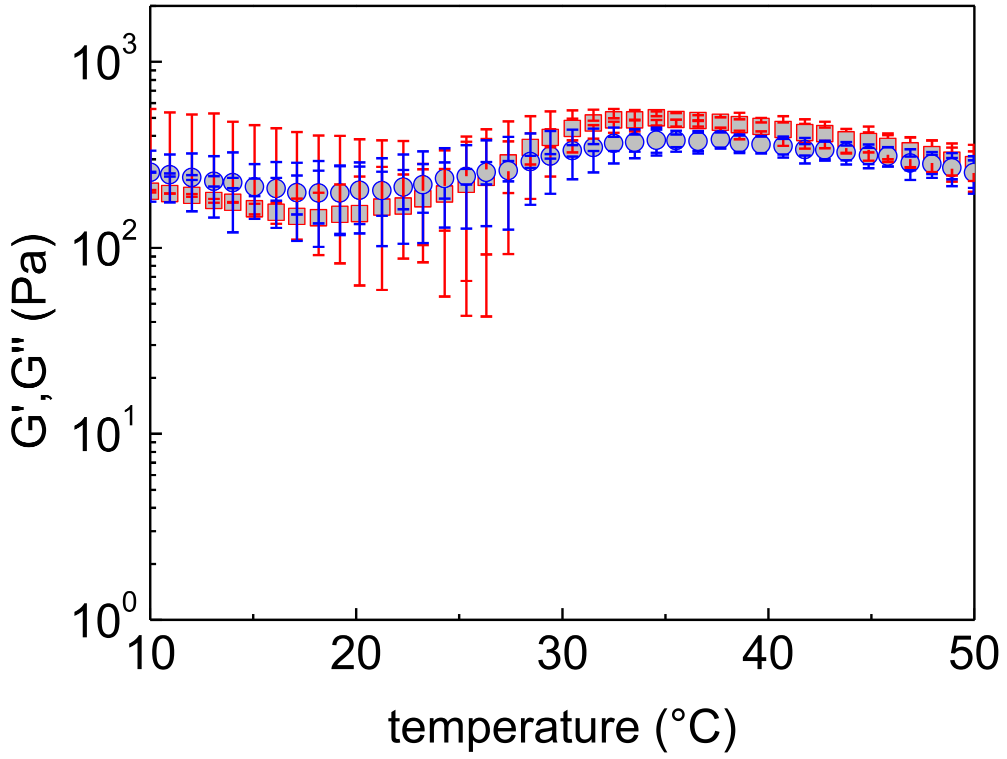

Figure S4. Storage modulus (red) and loss modulus (blue) as function of temperature of 5 wt.% 70/30 SIPN hydrogels at frequency 1 Hz, strain amplitude 0.1% and during the heating cycle with heating rate of 1 °C/min; Error bars indicate the reproducibility of the network as the rheological measurements have been conducted by three different replicate 70/30 hydrogels.

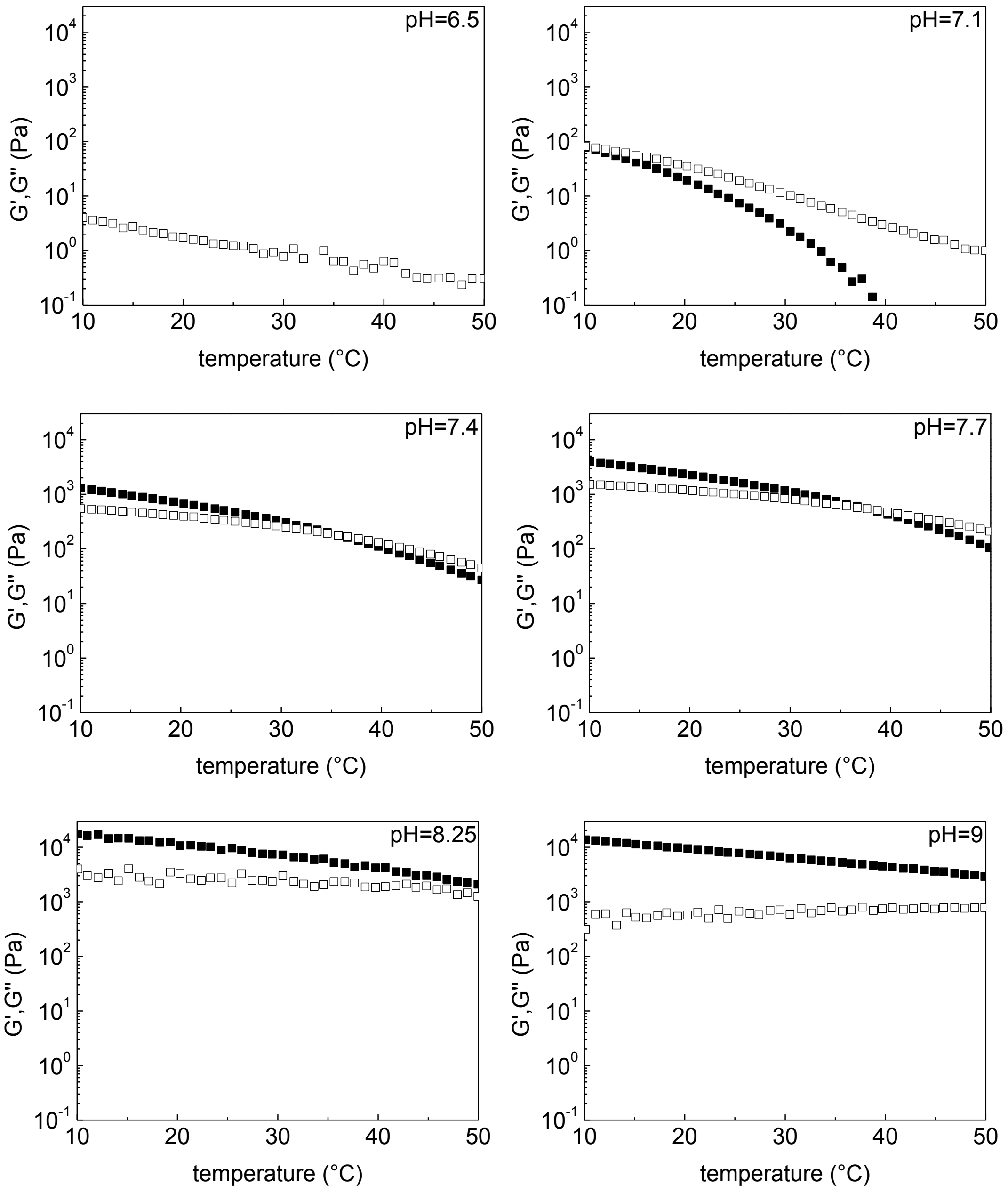

Figure S5. G’ (closed) and G’’ (open symbols) as function of temperature of 5 wt.% NaALG-g BA hydrogels at various pH. The experiments were performed at 6.28 rad/s, strain amplitude 0.1% and at heating rate of 1 °C/min.

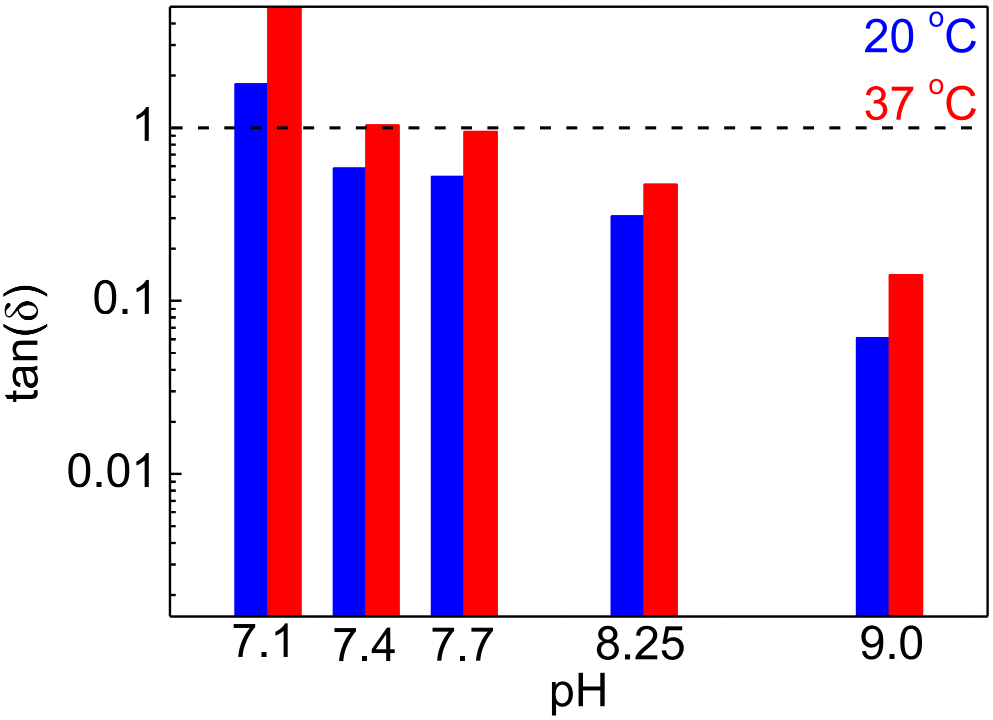

Figure S6. Tan(δ) as function of pH values of 5 wt.% NaALG-g-BA hydrogels at 20 °C and 37 °C.

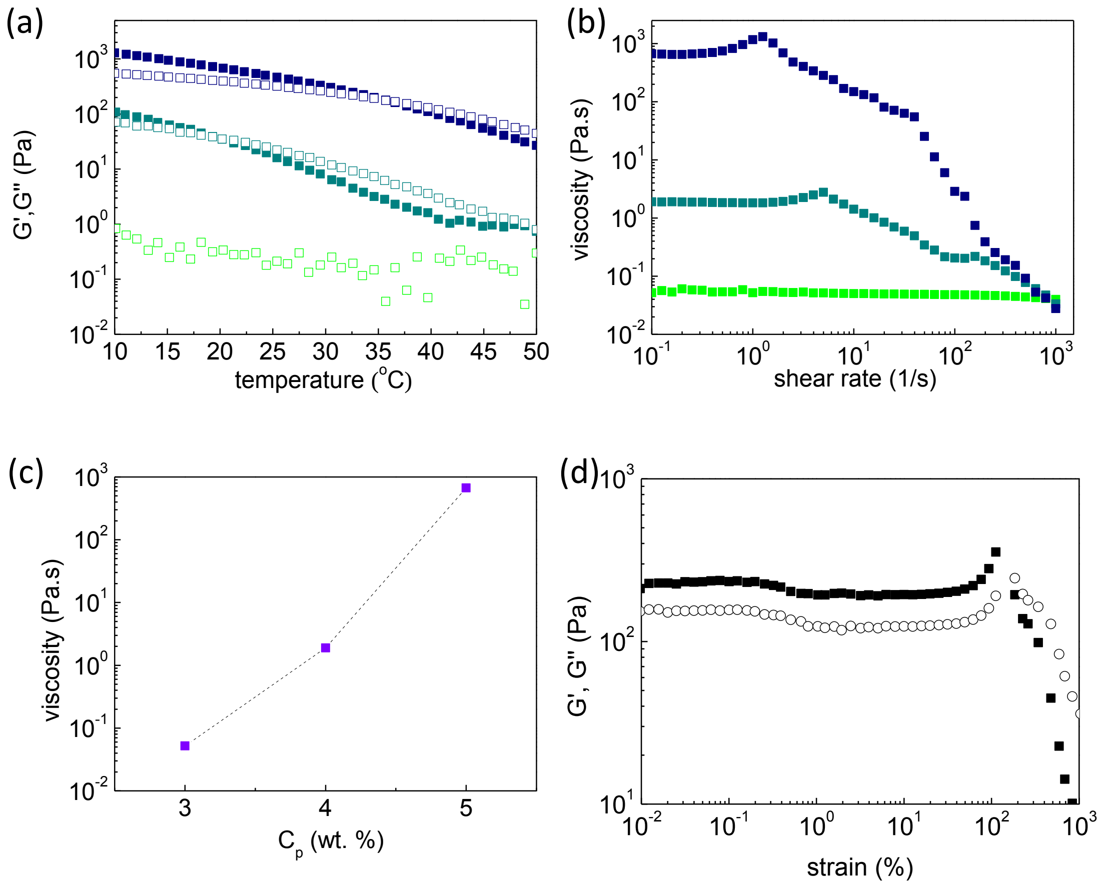

Figure S7. a) Storage (closed) and loss (open) moduli versus temperature of 5 wt.% (blue), 4 wt.% (cyan), 3 wt.% (green) NaALG-g-BA hydrogels at physiological pH,

6.28 rad/s, strain amplitude 0.1% and at heating rate of 1 °C/min; b) viscosity as function of shear rate of the previous described hydrogels; c) viscosity as function of polymer concentration at low shear rate at 10^{-1} s^{-1} at 20 °C; d) G' (closed) and G" (open) as function of strain (%) of 5 wt.% NaALG-g-BA at 20 °C and at 6.28 rad/s.

Injectability

As previously reported, the limit of the injection force F for a comfortable injection is 12 N[3]. F is given by equation (2), where η is the shear viscosity and Q_v the flow rate of a liquid injected through a syringe of radius R_s, needle of radius R_n, and length L, with F_f the friction force of the piston in the syringe[4].

$$F = \frac{8\eta L Q_v R_s^2}{R_n^4} + F_f \quad (2)$$

For a 27G syringe: R_s=2.4 mm, R_n=0.105 mm, L=12.7 mm. Considering that the friction force is negligible (F_f=0) and applying Q_v=1mL/min, equation (2) can be written as F=Kη where K=80.24 m^2 s^{-1}

For a comfortable injection, F should be lower than 12 N[1]. Therefore, the shear viscosity should be lower than η=F/K=12 N/80.24 m^2 s^{-1}=0.15 Pa.s.

Moreover, the shear rate $\dot{\gamma}$ applied during injection can be calculated by equation (3).

$$\dot{\gamma} = \frac{4Q_v}{\pi R_n^3} \quad (3)$$

For a 27G syringe (R_n=0.105 mm) and Q_v=1mL/min $\dot{\gamma}$=18.35 x 10^3 s^{-1}.

REFERENCES

[1] C. Barnett, F. Joubert, A. Iliopoulou, R. S. Álvarez, G. Pasparakis, *Mol. Pharm.* **2023**, *20*, 1818.

[2] T.-C. Chou, *Cancer Res.* **2010**, *70*, 440.

[3] T. E. Robinson, E. A. B. Hughes, A. Bose, E. A. Cornish, J. Y. Teo, N. M. Eisenstein, L. M. Grover, S. C. Cox, *Adv. Healthc. Mater.* **2020**, *9*, 1901521.

[4] A. Allmendinger, S. Fischer, J. Huwyler, H.-C. Mahler, E. Schwarb, I. E. Zarraga, R. Mueller, *Eur. J. Pharm. Biopharm. Off. J. Arbeitsgemeinschaft fur Pharm. Verfahrenstechnik e.V* **2014**, *87*, 318.
